# Biomechanical adaptation to post-stroke visual field loss: a systematic review

**DOI:** 10.1186/s13643-021-01634-4

**Published:** 2021-03-27

**Authors:** Adel Elfeky, Kristiaan D’Août, Rebecca Lawson, Lauren R. Hepworth, Nicholas D. A. Thomas, Abigail Clynch, Fiona J. Rowe

**Affiliations:** 1 Department of Health Services Research, University of Liverpool, 1-3 Brownlow Street, Liverpool, L69 3GL UK; 2 Institute of Life Course and Medical Sciences, University of Liverpool, Liverpool, UK

**Keywords:** Biomechanical, Movement, Hemianopia, Stroke, Visual field

## Abstract

**Background:**

Homonymous visual field defects represent the most frequent type of visual field loss after stroke, affecting nearly 30% of individuals with unilateral post-chiasmal brain damage. This review aimed to gather the available evidence on the biomechanical changes to visual field loss following stroke.

**Methods:**

A systematic review was conducted inclusive of randomised controlled trials, cohort studies, before-after studies and case-controlled studies. Studies including adult and paediatric participants that investigated eye, head, or body movements in post-stroke visual field loss during visual exploration tasks were included. Search terms included a range of MESH terms as well as alternative terms relating to stroke, visual field loss, hemianopia, visual functions and scanning behaviour. Articles were selected by two authors independently. Data were extracted by one author and verified by a second. All included articles were assessed for risk of bias using checklists appropriate to the study design.

**Results:**

Thirty-six articles (1123 participants) were included in the overall review (Kappa 0.863) and categorised into simulated or true visual field loss (typically hemianopia). Seven studies identified the biomechanical alterations to simulated hemianopia compared to normal performance. Twenty-nine studies detailed eye, head and body movement parameters in true hemianopia. Hemianopic participants and healthy adults with simulated hemianopia differed significantly from controls in various fixation and saccade parameters as indicated by increased number and duration of fixations, number and duration of saccades and scan path length with shorter mean saccadic amplitude. Under simulated hemianopia, participants were consistently biased towards the sighted visual field while gaze behaviour in true hemianopia was biased in the direction of the blind hemifield.

**Conclusions:**

There is considerable evidence on the altered eye movements that occur in true hemianopia and in healthy adults with simulated hemianopia. Successful performance in naturalistic tasks of visual exploration appears to be related to compensatory mechanisms of visual exploratory behaviour, namely, an increase in the amplitude and peak velocity of saccades, widening horizontally the distribution of eye movements, and a shift of the overall distribution of saccades into the blind field. This review highlights the lack of studies reporting head and other body movement parameters in hemianopia. Further studies with robust methodology and large sample sizes involving participants with post-stroke visual field loss are needed.

**Systematic review registration:**

PROSPERO CRD42020194403

**Supplementary Information:**

The online version contains supplementary material available at 10.1186/s13643-021-01634-4.

## Background

Stroke affects approximately 100,000 persons per annum in the UK [1]. As stroke is more common with older age, and the UK population is one of many countries with an ageing population, stroke is likely to be an ongoing health concern. A common problem post-stoke is visual impairment, with an estimated 65% of stroke survivors having visual impairment in the immediate aftermath of stroke [2]. The large prevalence of sight loss post-stroke imposes significant costs on public funds, private expenditure and health: an estimated £28.1 billion in 2013 in the UK [3].

Stroke-associated visual impairment can include impairment to central vision and peripheral vision (visual field), eye movement disorders, reading difficulties and visual perception disorders including visual neglect [2, 4]. Visual field defect (VFD) encompasses hemianopia, quadrantanopia, temporal crescent defect and scotoma, among others, with the most common defect being homonymous hemianopia (HH). HH involves vision loss on the same side of the visual field in both eyes and is associated with a worse prognosis for successful rehabilitation [5, 6], especially when combined with visual neglect [7]. Approximately 30% of stroke survivors have this visual field loss acutely while approximately 8–10% of stroke survivors have a permanent HH [8].

HH seriously impacts functional ability and quality of life following stroke [9]. For example, it causes an increased risk of falling, impaired ability to read, poor mood and higher levels of institutionalisation [10, 11]. Moreover, HH impacts participation in post-stroke rehabilitation and may result in poor long-term recovery, leading to loss of independence, social isolation and depression [12].

Individuals with VFD cannot process images in the same way as those with a full visual field [13]. Those with normal visual function use their peripheral field of vision as a guidance system and cue for generating eye movements to look towards objects of interest. To attain the highest quality of visual information, one must position the area of interest on the fovea [14–16]. Gaze scanning can be accomplished through eye, head and body movements, with the choice of movement depending on the demands of the activity and the environment. For example, when switching visual attention from one target to a closely adjacent target, the task usually requires only small eye movements. Conversely, when crossing the street, a task that involves acquiring information over a large area, individuals prefer to make a head movement to increase the scan area in a short period. This occurs because, in an outdoor environment, about 85% of naturally occurring human saccades have magnitudes of ≤ 15° [17]. Head movements also serve to recentre the head on the torso and serve as a reference frame for body movements, important for walking and maintaining consistent heading direction [18].

Individuals with normal or corrected-to-normal vision always move their eyes during the acquisition of visual information, even when attempting to maintain a steady gaze on a single point. Rapid gaze shifts known as saccades typically occur 2–3 times per second, bringing a new portion of the visual scene on to the fovea. In between these movements, the eyes essentially stop scanning about the scene, holding the central foveal vision in place so that the visual system can take in detailed information about what is being looked at [16, 19, 20]. Impaired peripheral vision (as occurs in hemianopia), will affect the visual feedback system that guides and cues eye movements into the affected visual field [21, 22]. Those with HH receive no visual cues from peripheral vision as to when to scan or how far to scan into the blind hemifield (BHF) and spend most of their time looking towards their BHF when viewing simple patterns in order to bring more of the visual scene into their seeing hemifield (SHF). They demonstrate numerous refixations (additional eye movements) and inaccurate saccades which result in impaired scanning, longer search times (trying to find objects) and the failure to detect relevant objects [23–26].

Training programmes exist to improve these eye movements—visual scanning or visual search strategies [27]. Research on the impact of HH on eye movements plus research on improving eye movements through visual scanning training is largely based on computerised tasks with participants seated in head-fixed positions [28]. While more recent research includes studies with free head movement, there has been limited investigation regarding the impact of hemianopia on eye movements when individuals are walking around with free head and trunk movements [29].

### Why this review is important

In real-life settings, some stroke survivors with HH spontaneously adapt to their visual field loss through effective compensatory eye and head movements and, within weeks of their stroke onset, can read easily, negotiate familiar and unfamiliar environments and appear to have little detriment to their everyday activities. Others appear to be more affected by visual field loss and continue using ineffective scanning strategies. This causes difficulties when carrying out daily activities such as reading, driving and locating objects around them [30]. It is not fully understood why some individuals adapt at a different rate to others using compensatory eye and head movements. Those who adapt well have a noticeable improvement in activities of daily living over those who do not. An important consideration for understanding the scanning performance is the extent to which individuals with these visual field defects might adopt patterns of eye and head movements that assist them to compensate to their visual impairment. The review will outline these compensatory strategies and provide indications as to whether some of these scanning patterns could be trained in rehabilitation programmes with the aim of improving visual search performance in this population.

To our knowledge, no systematic review has attempted to collate the available evidence on biomechanical alterations in stroke survivors with HH.

## Aim

To provide a comprehensive systematic overview of the biomechanical alterations to post-stroke VFD to identify which movement parameters are the most relevant, commonly used or have specific clinical relevance. This will guide current practice and aid in the design of future research into this subject area. In this review, the term biomechanics refers to changes in eye, head or body movements in response to the visual field loss.

### Objectives

The primary objective was to determine how eye, head and body movements are affected by the visual field loss that occurs following a stroke compared to healthy controls and participants with simulated VFD.

The secondary objectives were to determine how biomechanical factors are affected by extent and side of visual field loss.

## Methods

This systematic review aimed to bring together the biomechanical evidence relating to eye, head and body movements in stroke-related VFD. The review was observed and reported according to the PRISMA (Preferred Reporting Items for Systematic Reviews and Meta-Analyses) guidelines (**Supplementary Figure** 1) [31]. A detailed protocol was developed prior to the review and registered with PROSPERO (international prospective register of systematic reviews; CRD42020194403) [32].

### Inclusion criteria for considering studies for this review

#### Types of studies

The following types of studies were included in the review: randomised controlled trials, controlled trials, prospective cohort studies, before-after studies, case-controlled studies and case series. Articles that discussed other visual impairments alongside VFD were included if visual field loss was discussed separately. Interventional studies for visual field loss were included provided that they formally recorded eye, head or body movements. Studies that only recorded behavioural outcomes (for example, reading) to assess the effectiveness of an intervention were excluded. Case reports and letters were excluded due to the risk of bias associated with these types of reports. All languages were included and translations were obtained when necessary.

#### Participants

We included studies of adult participants of all ages and children diagnosed with post-stroke VFD. Studies which included mixed populations were included if over 50% of the participants had a diagnosis of stroke and data were available for this subgroup. Studies which included participants with HH and other VFD were included if over 50% of the participants had hemianopia and data were available for this subgroup. Studies that included participants with both HH and neglect were also included if HH data could be extracted separately to that of HH with neglect or neglect only.

### Target condition

The target condition was visual field loss of any severity which occurred acutely following a stroke event, and simulated HH.

### Study tasks

Studies that investigated the compensatory eye, head or body movements to post-stroke VFD or simulated HH during visual exploration tasks were included. Studies that investigated practice-related changes in movement strategy were also included provided that eye, head or body movements were quantitatively measured using kinematic equipment (e.g. eye trackers).

### Comparator(s)/control

Eye, head or body movements in individuals with VFD were compared to other groups (i.e. individuals with simulated HH and normally sighted participants).

### Outcome measures

Eye, head or body movement parameters expressed in quantitative data during different task types: Percentage change or difference in measurement from baseline to primary endpoint or before/after intervention for; number of fixations, refixations, saccades, saccade amplitude, proportion of saccades made into the BHF and SHF, head turn, shoulder movement, etc.

### Information sources and search strategy

We used systematic search strategies to search key electronic databases. The following electronic databases were searched: PsycINFO (1887 to 2020), Scopus (1823 to 2020), MEDLINE (1948 to 2020), CINAHL (1937 to 2020). No language restrictions were applied. We also hand-searched the reference lists of all included studies for relevant papers. Search terms are detailed in Table 1.

**Table 1:** Search terms

Cerebrovascular disorders Brain ischaemia / Intracranial Arterial Disease/ Arteriovenous Malformations/ Intracranial Embolism and Thrombosis/ Stroke/	Hemianopia/ Visual Fields/ Eye/ Eye Disease/ Visually Impaired Persons/ Vision Disorders/ Blindness/ Vision, Binocular/ Vision, Monocular/
OR	OR
AND	

### Data management

References from all searches were uploaded into EndNote (X9, Clarivate Analytics, USA) bibliographic software. Duplicates were removed using the Endnote deduplication tool.

### Selection process

The titles and abstracts identified from the search were independently screened by one reviewer (AE) and at least 10% were double checked by a second author (AC) using the pre-stated inclusion criteria. A secondary review of the full papers was then undertaken independently by two reviewers (AE reviewed all studies along with either KD, RL, NT, or FR) and a Cohen’s Coefficient of Agreement [33] (Kappa score) calculated to quantify inter-reviewer agreement. Any disagreements over inclusion of studies were discussed and resolved by discussion between all reviewers.

### Data extraction process

A pre-designed data extraction form was used to gather information on study design, aims, sample size, numbers recruited and analysed, intervention and outcome measures related to eye/head/body movement. Data was extracted and documented by one reviewer (AE) and verified by a second (either KD, RL, LH, NT or FR). Data was reported by numbers, means, standard deviation (SD) and standard error of mean (SEM). Of note, measurement data are provided in the tables where available and *p* values when no hard data is reported in the primary studies.

### Quality assessment

Two reviewers (AE reviewed all studies along with either KD, RL, NT or FR) independently reviewed the quality of the studies included in this review using the following two checklists: (1) For the quality assessment of randomised control trials, the CONSORT (Consolidated Standards of Reporting Trials) statement was used. The CONSORT statement covers 25 items within the following domains; title/abstract, introduction, methods, results, discussion and other information [34].

(2) The STROBE (Strengthening the Reporting of Observational Studies in Epidemiology) statement was used to assess the quality of cohort, before-after and case-control studies. The STROBE statement covers 22 items from introduction, methods, results and discussion [35]. A study was considered of good quality if it scored 75% or greater on the relevant checklist.

## Results

**Fig. 1:**
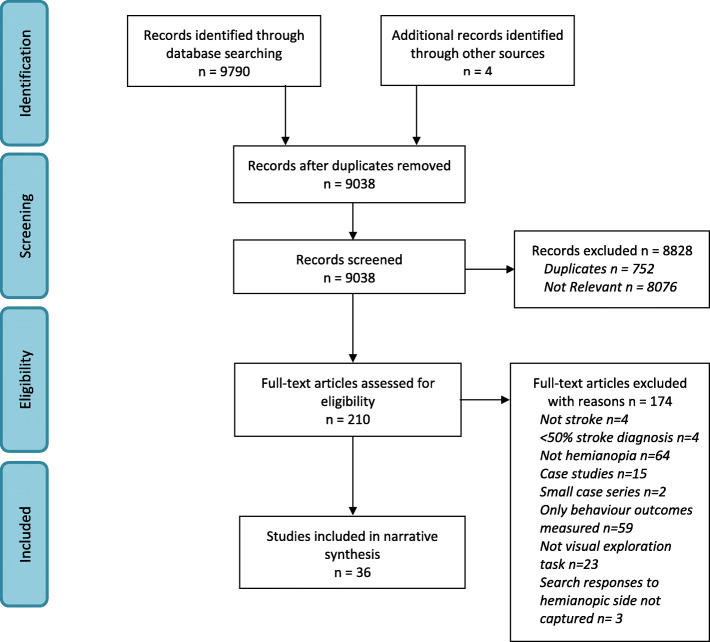
PRISMA flow diagram

Figure 1 illustrates the flowchart for this review. Thirty-six articles (1123 participants) published in English were included and the overall Kappa score for inter-rater agreement was 0.863 indicating substantial agreement. Due to the heterogeneity across the included studies with respect to reporting of outcomes as well as recruitment and selection of participants, a meta-analysis of studies could not be undertaken. A narrative summary of the data is presented in relation to included studies to highlight how visual field loss impacted on eye, head or body movements across the included studies. Results are split into simulated HH and true VFD and then discussed by type of movement analysis (eye/head/body movement). Data analysis was conducted by one reviewer (AE) and double checked by another two reviewers (FR, LH).

### Quality assessment

A total of 36 articles were included in this review paper and the risk of bias was assessed for each (**Supplementary Tables** 1**,** 2). Overall, no article scored 100% for quality assessment in this section. Twenty-nine of the 36 articles scored between 76 and 90% of the checklist items assessed and were deemed to have good quality. Seven studies scored between 59 and 71% on the relevant quality checklists. All articles were included in this review.

### Simulated hemianopia

Seven papers recruited healthy adult participants and simulated HH (*n* = 269 summed sample size). None of the included studies recruited children in their sample. These included the following study types; one randomised cross-over trial (*n* = 24), three cohort studies (*n* = 147), one case-control study (*n* = 34) and two before-and-after studies (*n* = 58). HH was simulated in these studies using a gaze-contingent visual display paradigm. When simulating HH, a window, with the same properties as the background, continuously and completely blanked part of the screen with reference to the gaze position. The blanked area could either be left or right of fixation in order to simulate left- or right-sided HH respectively. Table 2 summarises the key data extracted from the simulated HH studies.

**Table 2:** Key data extracted from simulated hemianopia studies

Study ID	Study design	Sample size	Population	Intervention(s)	Tasks	Type of movement formally recorded (eye /head/ body)	Outcome measures	Results summary
Liman et al. (2012) [36]	Cohort study	*n =* 20	Adult subjects with no neurological or psychiatric disorders	N/A	Subjects’ eye movements were recorded under simulated HH condition during viewing and subsequent visual imagery of complex pictures	Eye movements	Saccade amplitude, number of fixations, fixation duration and global/ local ratio	Fixation duration was significantly higher in visual imagery scanpaths than in the viewing condition (session 1: 0.32 s viewing–0.39 s imagery (*p* < 0.001); session 3: 0.295 s viewing–0.34 s imagery). Number of fixations was lower in visual imagery scanpaths than in the viewing condition (session 1: 12.4 viewing–9.8 imagery (*p* < 0.001); session 3: 12.5 viewing–9.9 imagery (*p* < 0.001)). Saccade amplitudes were significantly lower in visual imagery scanpaths than in the viewing condition (session 1: 2.7° viewing–2.4° imagery (*p* = 0.05); session 3: 3.15° viewing–2.3° imagery (*p* < 0.016)). Global/Local ratio was significantly lower in visual imagery scanpaths than in the viewing condition (session 1: 8.3 viewing - 6.3 imagery (*p* = 0.04); session 3: 11.25 viewing - 6.1 imagery (*p* < 0.001)).
Nowakowska et al (2016) [37]	Cohort study	*n =* 101	Adult subjects with normal or corrected-to-normal vision	N/A	Eye movements were recorded while participants searched for an emotional face among neutral faces	Eye movements	Number of fixations, saccade amplitude, proportion of saccades into the blind field	In masked conditions (blank, dot, filtered),an increased number of fixations was found (22,20,15 respectively) compared to unmodified condition (13). No significant difference in saccadic amplitude between the saccades made into the sighted hemifield (SHF) and blind side (BHF) in the unmodified condition (*p* = 0.87). In masked conditions, saccades were shorter when made towards the SHF compared with the BHF (all *p* values < 0.002). The proportion of all saccades made into the BHF was significantly smaller in the blank (M = 0.44, SD = 0.11, *p* = 0.001), dot (M = 0.42, SD = 0.07, *p* < 0.001), and filtered (M = 0.47, SD = 0.06, *p* = 0.05) conditions compared with unmodified (M = 0.52, SD = 0.04)
Nowakowska et (2019) [38]	Case-control study	*n =* 34	Adult subjects with normal or corrected-to-normal vision	Participants practised three visual search tasks (repeated search task, object naming and detection task) in five testing sessions over five consecutive days	N/A	Eye movements	Reaction time, eye movement behaviour in the repeated exposure task	Simulated HH slowed search but improved over the five sessions. Most participants improved on the task later on in the week by shifting their fixations into the BHF (*p* = 0.002).
Schuett et al (2009a) [39]	Cohort study	*n =* 32	Adult subjects with normal or corrected-to-normal vision	N/A	Eye movements were recorded during manual and ocular line bisection tasks	Eye movements	Fixation range, number and duration of fixations, number and amplitude of saccades and scanpath length	During manual line bisection, the number of right-hemispace fixations in RHH (58.2 (21.9)) was higher than controls (9.89 (9.9)) and fixation duration was higher (453.7 ms, 419.2 ms respectively). Left-hemispace fixations in LHH (48.3 (23.3)) were higher than controls and fixation duration significantly increased (560.7 ms, 493.5 ms respectively). Participants made larger saccades towards the BHF than towards the SHF (*p* < 0.017). The spatial range covered by fixations was considerably larger (LHH: 12.0° (4.9), RHH: 13.3° (4.2)) than normal viewing (6.9° (5.8)), scanpaths significantly longer (LHH: 191.5° (113.1), RHH: 229.1° (89.6)) than under normal viewing conditions (50.0° (49.0)). During ocular line bisection, the number of right-hemispace fixations in RHH (38.2 (19.6)) was higher than normal viewing (16.9 (9.8)) and fixation duration was higher (316.2 ms (92.0), 298.8 ms (109.5) respectively). Left-hemispace fixations in LHH (40.7 (19.6)) were higher than normal viewing (21.5 (15.8) and fixation duration slightly increased (366.1 ms (175.6), 353.7 ms (157.8) respectively). Scanpaths were slightly longer (LHH: 295.2° (168.1), RHH: 290.6° (181.2)) than under normal viewing conditions (226.0° (121.4)).
Schuett et al (2009b) [40]	RCT	*n =* 24	Adult subjects with normal or corrected-to-normal vision	15-min session of reading and visual exploration practice	Eye movements were recorded during reading and visual exploration	Eye movements	Mean reading time, number of fixations, fixation duration, repeated fixations (%), mean number and amplitude of forward saccades, scanpath length	During visual exploration, exploration times increased, participants made many more errors and the prolonged scanpath was characterised by a higher number and duration of fixations. However, participants failed to show the expected decrease in return sweep and exploration saccadic amplitude (*p* = 0.126). After visual exploration practice, participants showed a significant decrease in the number of fixations (pre: 30; post: 24), and scanpath length (pre: 125°, post: 75°). Fixation duration decreased slightly after training (380 ms) compared to pre-training (460 ms).
Schuett et al (2009c) [41]	Before-after sudy	*n =* 37	Adult subjects with normal or corrected-to-normal vision	Participants performed two visual exploration practice sessions (15 min each)	Eye movements were recorded during visual exploration	Eye movements	Number and mean duration of fixations, number and mean amplitude of forward saccades, mean amplitude of return sweep saccades	After visual exploration practice, number of fixations significantly reduced (LHH, pre: 24.0, post: 19.8; RHH, pre: 26.8, post: 21.1) with shorter fixation durations (LHH, pre: 463 ms, post: 407 ms; RHH, pre: 449 ms, post: 381 ms ). There was no significant effect for overall, left- and rightward saccadic amplitude; practice did also not affect number and duration of left- and right-hemispace fixations (*p* = 0.141).
Zangemeister et al (2002) [42]	Before-after study	*n =* 16	Adult subjects with normal or corrected-to-normal vision	Eight subjects (“early training group”) were trained for 20 min with 10 deg off-field defect towards the healthy side and directly afterwards. The other 8 subjects (“late training group”) underwent 20 min of viewing without any training.	Eye movements were recorded while a series of 2 × 8 pictures were presented during simulated HH.	Eye movements	Fixation durations, saccadic amplitudes, ratio of overshoot one-step/ undershoot stair-step saccades to the BHF	For the early training group, fixation durations decreased significantly immediately after training (0.26 s) and 14 days afterwards (0.39 s) compared to pre-training (0.43 s). Saccadic amplitude increased significantly immediately after training (6.2°) and 14 days afterwards (4.9°) compared to pre-training (3.4°). Ratio of overshoot one-step/ undershoot stair-step saccades to the BHF significantly increased immediately after training (0.76) and 14 days afterwards (0.52) compared to pre-training (0.37). For the late training group, fixation durations significantly decreased 14 days after training (0.29 s) compared to pre-training (0.41 s). Saccadic amplitude significantly increased 14 days post training (5.8°) compared to pre-training (3.5°). Ratio of overshoot one-step/ undershoot stair-step saccades to the BHF also significantly increased 14 days post training (0.67) as compared to pre-training (0.36).

*BHF* blind hemifield, *SHF* sighted hemifield, *HH* homonymous hemianpia, *LHH* left homonymous hemianopia, *M* mean, *RHH* right homonymous hemianopia, *SD* standard deviation

None of these simulation studies assessed either head or body movement; therefore, this section will focus solely on eye movements. Eye movements were recorded in three studies using a pupil and dual Purkinje image video eye-tracker [39–41]. In two studies, eye movements were recorded using pupil-corneal-reflection method [37, 38]. Two studies recorded eye movements with infrared reflection oculography [36, 42].

#### Eye movement parameters

##### Number, duration of fixations and percentage of fixation repetitions

The most commonly reported eye movement measurements included number of fixations, duration of fixations and percentage of fixation repetitions. These eye movement parameters were measured in six of the seven studies (*n* = 203). During visual tasks with simulated HH (visual exploration, line bisection), participants showed significantly more and longer fixations and refixations when compared with their normal performance [37, 39–41]. However, eye movement recordings during tasks that require complex cognitive processing (e.g. visual mental imagery of complex pictures) revealed that participants made significantly fewer fixations compared to normal viewing of stimuli [36]. Participants with simulated HH also changed their oculomotor search strategy in response to increasing search difficulty as demonstrated by a significantly decreased fixation duration for detailed tasks (to memorise the details of each picture) and recollection tasks (to look for relationships between items) compared to easy tasks (to look at the whole picture) [36].

Efficient spontaneous oculomotor changes to simulated HH occurred after 15 min of visual exploration practice [40]. Improvements in visual exploration mirrored changes of the respective oculomotor measures as participants showed a significant decrease in the number and duration of fixations. Zangemeister et al. [42] also demonstrated that training of parafoveal eccentric viewing for 20 min helped the oculomotor change of participants with simulated HH significantly. Fixation durations decreased significantly immediately after training and 14 days later compared to pre-training.

##### Saccade amplitude and scan path length

Saccadic amplitude (i.e. the angular distance travelled by the eye between two fixation points) and scan path (i.e. the sum of all saccadic amplitudes) eye movement parameters were measured in six studies (*n* = 235). Scan paths were significantly longer across various visual search tasks with simulated HH [39–41]. The amplitude of saccades, however, varied significantly and was task dependent. Eye movement recording during viewing and subsequent visual imagery of complex pictures revealed that saccade amplitudes were significantly lower in visual imagery scan paths compared to the normal viewing condition [36]. During visual exploration, however, participants did not show the expected decrease in return sweep and exploration saccadic amplitude [39]. In a study of visual search for an emotional face among neutral faces, no significant differences in saccadic amplitude were found between the saccades made into the SHF and BHF in the unmodified condition. However, in the masked conditions (blank/grey background), dot (black and white dots representing the spatial location of the faces) and filtered (the low spatial frequency version of the face), saccades were shorter when made towards the SHF compared with the BHF [37].

Practice-related changes in saccadic amplitude and scan path length under simulated HH were found. After visual exploration practice, participants showed a significant decrease in scan path length [40]. Participants undergoing training of parafoveal eccentric fixation also showed a significant increase in saccadic amplitude immediately after training and 14 days later compared to pre-training [42].

##### Proportion of saccades into the blind hemifield

As a measure of directional bias, three studies (*n* = 167) examined the mean proportion of all saccades executed towards the BHF. In various visual tasks (object naming, detection and search) [37, 38], participants with simulated HH were consistently biased towards the visible part of their visual field. Participants preferred to saccade first, and more often, into the SHF as opposed to the BHF, irrespective of task difficulty. The bias to preferentially search the sighted field persisted even in easy search. Liman et al recorded eye movements during viewing and subsequent visual imagery of complex pictures [36]; under masked conditions, the proportion of all saccades made into the BHF was significantly smaller in the blank, dot and filtered conditions compared with the unmodified condition . With practice, most participants made saccades that went further into the BHF and earlier in the search process, specifically under conditions where little information about the target location would be gained by inspecting the SHF. Contrary to the search required for a full-view environment, participants made significantly larger saccades towards to their BHF during line bisection than into their SHF when compared to normal viewing conditions [41]. In participants with left HH (LHH), the amplitude of leftward saccades was significantly higher than that of rightward saccades. In participants with right HH (RHH), the amplitude of rightward saccades was significantly higher than that of leftward saccades.

##### Global/local ratio

Global/local ratio (g/l) is a measure to distinguish between global versus local viewing strategies (saccades smaller than 1.0° were taken as local and those larger than 1.1° were taken as global). The g/l ratio was measured in one study (*n* = 20) that examined the impact of simulated HH on scan path eye movements during visual mental imagery [36]. It was reported to be significantly lower with simulated HH when compared to eye movements during regular viewing of stimuli.

Significant effects of tasks and picture content on fixation g/l ratio were also found. Participants demonstrated a significantly increased median g/l ratio for abstract and realistic picture content. The highest g/l ratio was detected for abstract picture content and for a detailed viewing task. The task effect in the “detailed” condition elicited a more global scanning of the whole image. This effect was stronger in abstract than in realistic or search pictures reflecting in part the visual content of the inspected image.

##### Ratio of overshoot one-step/undershoot stair-step saccades to the blind hemifield

The ratio of overshoot one-step/undershoot stair-step saccades to the BHF was recorded in one study (*n* = 16) as a measure of efficient adaptation [42]. The ratio was significantly higher 14 days after training of parafoveal eccentric viewing as compared to pre-training. This is an indicator of efficacy in improving gaze by use of saccades of larger amplitudes towards the BHF.

#### Head movements

None of the simulated hemianopia studies measured head movements.

#### Shoulder movements

None of the simulated hemianopia studies measured shoulder movements.

##### Summary of key findings from simulated hemianopia studies

Simulated HH induced an inefficient and unsystematic oculomotor scanpath for exploring and processing visual information during visual exploration tasks, as indicated by the increase in number and duration of fixations as well as in scan path length. Participants preferred to saccade first, and more often, into the SHF as opposed to the BHF to a similar extent across various difficulty levels. During tasks that required complex cognitive processing and under masked conditions, saccade amplitudes were significantly lower compared to normal viewing and the proportion of all saccades made into the BHF was significantly smaller.

After visual exploration training, however, participants developed a more efficient oculomotor response to the visual-sensory loss which improved their visual exploration performance. Participants showed a significant decrease in the number and duration of fixations and scan path length. Over time, participants scanned further into their BHF than into their SHF and made significantly larger saccades towards the side of space corresponding to their BHF.

### True hemianopia

Twenty papers recruited adult participants with VFD (*n* = 397 summed sample size) and healthy controls (*n* = 284). Nine further studies recruited only participants with VFD (*n* = 173). None of the included studies recruited children in their sample. Only two studies included participants with both HH and visual neglect but reported results separately for these groups. Together these papers included the following study types; two randomised control trials (*n =* 50), five before-after studies (*n =* 153), 18 case-control studies (*n =* 632), three cohort studies (*n =* 45) and one case series (*n =* 6). Eye movements were recorded in 27 studies (*n =* 846) and eight studies (*n =* 221) recorded head or body movements. Only two studies (*n =* 100) recorded eye, head and shoulder movements. Table 3 summarises the key data extracted from the real VFD studies.

**Table 3:** Key data extracted from true hemianopia studies

Study ID	Study design	Sample size	Aetiology	Population (adults/children)	Intervention(s)	Tasks	Type of movement formally recorded (eye /head/ body)	Outcome measures	Results summary
Alberti et al (2017) [43]	Cohort study	*n =* 12 HH (6 right, 6 left)	Stroke (*n =* 8) Tumour (*n =* 3) Trauma (*n =* 1)	Adults	N/A	Participants completed two sessions in a driving simulator pressing the horn when they detected a pedestrian	Eye movements	Gaze and pedestrian eccentricity on the BHF. Gaze and pedestrian eccentricity on the SHF	On the blind side, the first scan to reach the pedestrian occurred later, on average, than on the seeing side (medians of 1.25 and 0.75 s, respectively). On the blind side only 40% of scans to reach the pedestrian were made within 1 s of the pedestrian appearing compared with approximately 70% on the seeing side. Median scan magnitudes did not differ between the two conditions (approaching: 14° (IQR 9°–15°); stationary: 13° (IQR 9°–20°); *p =* 0.43).
Bahnemann et al (2015) [44]	Case-control study	*n =* 41 HH	Ischemic infarction (*n =* 41)	Adults	N/A	Participants were tested in a fixed-base driving simulator and 3 driving scenarios were constructed.	Eye and head movements	Amplitude and the peak velocity of saccades, distribution of fixations, number and the duration of saccades, duration of fixations The number, amplitude, duration and the peak velocity of head movements	The low performance (LP) group showed a significantly smaller amplitude than either the control group (*p =* 0.031) or the High performance (HP) group (*p =* 0.030). The LP group also showed a significantly lower velocity than the HP group (*p =* 0.031), and the control group (*p =* 0.055). The LP group showed a significantly more narrow spread than either the control (*p =* 0.015) or HP group (*p =* 0.003), with the HP group showing the widest spread. The HP group displayed the highest percentage of fixations in the BHF (*p =* 0.002). No statistically significant differences were found concerning the number (*p =* 0.304) and the duration of saccades (*p =* 0.221). No difference regarding the duration of fixations (*p =* 0.567) between the healthy controls and the participant subgroups. No statistically significant differences were found regarding the number (*p =* 0.877), the amplitude (*p =* 0.696), the duration (*p =* 0.495), and the peak velocity (*p =* 0.350) of head movements between the two participant subgroups and the healthy controls.
Bowers et al (2014) [45]	Case-control study	*n =* 26 HH (*n =* 14, 8 left, 6 right) Normally sighted (*n =* 12)	Stroke (*n =* 11) Other (*n =* 2)	Adults	N/A	Using a driving simulator, the effects of HH on head scanning behaviours at intersections were examined	Head movement	Total number of scans, proportions of leftward scans, the magnitude of the leftward and rightward scans	No significant differences among the vision groups in the total number of scans (*p =* 0.253). The LHH group had a significantly higher proportion of leftward scans (76%) than normal controls and RHH groups (59% and 48%, respectively). The proportion of leftward scans was highest when there was an incoming road on the left only (74%) and lowest when there was no incoming road on that side (41%, *p <* 0.001). The RHH group made a significantly lower proportion of leftward head scans (i.e. a higher proportion of rightward scans) than normal controls and LHH drivers (*p =* 0.008 and *p <* 0.001, respectively) when there was no incoming road on their blind right side. LHH group made a significantly higher proportion of head scans to their blind left side than normal controls and RHH drivers (*p <* 0.001) when there was no incoming road on that side. For all groups, the magnitude of the leftward and rightward scans increased as distance to the intersection decreased (*p <* 0.001). Head scan magnitudes for the two HH groups were smaller than those of the control group (*p <* 0.001). For the HH groups, blind side scans were not larger than seeing side scans.
Cazzoli et al (2016) [46]	Case-control study	*n =* 32 Patient group (*n =* 24, 8 neglect, 6 quadrantanopia, 10 HH) Healthy controls (*n =* 8)	Ischemia (*n =* 17) Haemorrhage (*n =* 7) Healthy subjects (*n =* 8)	Adults	N/A	Participants freely explored a traffic scene without (static condition) and with (dynamic condition) naturalistic motion, i.e. cars moving from the right or left.	Eye movements	Mean gaze position during early attentional orienting. Cumulative fixation duration (CFD) spatial distribution (the sum of the duration of all visual fixations).	The mean gaze position during early attentional orienting was significantly rightward-deviated in participants with neglect and VFD in comparison to all other groups (i.e. participants with VFD only, participants with right-hemispheric lesions without neglect or VFD, and healthy subjects). Participants with neglect and VFD showed significantly lower percentage CFD values than healthy subjects. Participants with VFD showed significantly higher percentage CFD values than all other groups.
Fourtassi et al (2016) [47]	Cohort study	*n =* 19 9 patients (3 right HH, 2 left HH, 2 quadrantanopia, 2 no deficit). Two had HH + neglect; 2 had neglect but no VFD10 healthy subjects	Stroke (*n =* 9) Healthy subjects (*n =* 10)	Adults	N/A	Gaze positions were recorded during memory recall of French towns in an imagery task, a non-imagery task (verbal fluency), and a visually guided task.	Eye movements	Spatial consistency between gaze positions, spatial distribution of the gaze throughout the task, saccades’ directions and amplitudes.	Gaze was constantly shifted with respect to their body midline, contralesionally for all HH participants without neglect and ipsilesionally for the two HH participants with neglect. Participants performed a similar number of saccades of similar magnitude in both directions as did control subjects (rightward and leftward). No differences between the number or the mean magnitudes of rightward and leftward saccades, either in imagery or in non-imagery tasks (*p* > 0.05).
Gbadamosi et al (2001) [48]	Case-control study	*n =* 34 HH (*n =* 14, 7 left, 6 right, 1 bitemporal deficit) Healthy controls (*n =* 20)	Stroke, trauma, operation	Adults	N/A	Each subject was tested during a standardised routine that consisted of one stimulus presentation phase and three subsequent imagery phases. Six different stimuli that were all bordered by a reference frame were used.	Eye movements	Number of fixations, fixation duration, saccade amplitude	The number of fixations was lower in the imagery phases than the viewing scanpaths in both groups. Fixation duration was significantly higher in the imagery scanpaths than in the viewing scanpaths. The median saccade amplitudes were lower during the imagery phases in both groups.
Grasso et al (2016) [49]	Before and after study	*n =* 10 HH	Ischemic (*n =* 8) Haemorrhagic (*n =* 1) Arteriovenous malformation (*n =* 1)		Audio-visual training for 4 h daily, over a period of 2 weeks.	Eye movements were recorded during several visual tasks before and after audio-visual training	Eye movements	Exploration time, number of fixations, saccadic speed	Significant improvement in scanning efficiency. Number of fixations reduced: 80.9 to 73.6 to 70.2. Mean saccadic speed faster: 50.45d/s to 64.81d/s to 64.00d/s. Mean exploration time lower: 27.2 s to 23.5 s to 23.2 s.
Hardiess et al (2010) [50]	Case-control study	*n =* 24 Hemianopia (*n =* 10, 2 left, 8 right) Quadrantanopia (*n =* 2) Healthy controls (*n =* 12)	Ischemia (*n =* 10) Brain surgery (*n =* 1) Haemorrhage (*n =* 1)	Adults	N/A	All participants were tested in two tasks, i.e. a dot counting (DC) task requiring mostly simple visual scanning and a cognitively more demanding comparative visual search (CVS) task.	Eye and head movements	Task performance (response time), scanpaths, gaze performance, head movements	Search time significantly increased for the two participant groups (*p <* 0.001). No differences were found regarding the scanpaths between the normal subjects (230° DC, 1050° CVS) and the high performance group (240° DC, 1100° CVS). Low performance group performed with a highly increased number of fixations (34 DC, 52 CVS) repetition of fixations (9 DC) a higher proportion of fixations towards the impaired visual field (65% DC, 58% CVS) compared to controls (25 DC, 37 CVS / 52% DC, 53% CVS respectively). The scanpath appeared rather unsystematic and time-consuming (340°) compared to controls (230°). Participants also displayed significantly increased fixation duration (290 ms DC, 260 ms CVS) compared to controls (270 ms DC, 240 ms CVS ). The high performance group showed an organised scanning pattern similar to that of the unimpaired normal subjects. In the free head comparative visual search task all subjects performed maximum head movements in a range of ±3° and ± 20°. The average maximum amplitudes were larger for LP participants (14.13 ± 8.0 mean ± SD), while for HP participants they were within a range (8.89 ± 4.78 mean ± SD) similar to the one of normal subjects (9.12 ± 5.0 mean ± SD).
Kasneci et al (2014a) [51]	Case-control study	*n =* 40 HH (*n =* 10, 4 right, 6 left) Glaucoma (*n =* 10) Healthy controls (*n =* 20)	Stroke (*n =* 10) Glaucoma (*n =* 10)	Adults	N/A	Eye movements were recorded during a 40-min driving task	Eye, head and shoulder movements	Driving performance, horizontal gaze activity (HGA), horizontal gaze distribution (HGD), head and shoulder movements	Participants who failed displayed significantly reduced scanning activity in comparison to controls who passed the test (*p <* 0.05). Participants who failed the driving assessment performed significantly less exploratory head and shoulder movements than control subjects and participants who passed the driving test (*p <* 0.001). No difference was found regarding the horizontal gaze activity between the participant subgroups. Participants who passed the driving test performed significantly more glances towards their visual field defect than participants who failed (*p <* 0.05).
Kasneci et al (2014b) [52]	Case-control study	*n =* 20 HH (*n =* 10, 4 right, 6 left) Healthy controls (*n =* 10)	Ischemia (*n =* 8) Brain surgery (*n =* 1) Cranio-cerebral trauma (*n =* 1)	Adults	N/A	Eye movements were recorded while participants were asked to collect 20 products placed on two supermarket shelves as quickly as possible.	Eye movements	Horizontal gaze activity (HGA), glance proportion in percentage (GPP), glance frequency (GF)	No difference was found in HGA between the participant subgroups. No significant difference between the subject subgroups regarding the proportion of glances beyond the 30° VF (GPP30c). A significantly higher GPP-30° in participants with a right-sided defect compared with their controls. A significant difference in GPP beyond 60° (GPP-60c) between participants with left HH who passed (HHp) and participants with HH who failed (HHf). HHp performed significantly longer glances towards the visual field periphery beyond 60° than HHf (*p <* 0.05).
Keller et al (2010) [53]	RCT	*n =* 20 HH (*n =* 13, 7 right, 6 left) Quadrantanopia (*n =* 7)	Stroke (*n =* 18) Tumour (*n =* 1) Trauma (*n =* 1)	Adults	Audio-visual exploration training (AV) versus visual exploratory training (VT). Each participant received 20 therapy sessions (each session lasting 30 min) over 3 weeks.	Eye movements were recorded after training	Eye movements	Number of saccades, amplitude of saccades	Comparisons between the two forms of training revealed a significantly greater improvement for all outcome variables for the AV group. In particular AV stimulation significantly increased the number (56 AV and 37 VT) and amplitude of saccades (52° AV and 44° VT).
Lévy-Bencheton et al (2015) [54]	Before and after study	*n =* 14 HH (8 right, 6 left)	Ischemic stroke	Adults	The training consisted of three different anti-saccades (AS) training: one with (adaptation) and two without (delayed shift and no shift) saccadic adaptation. Each training lasted around 15–20 min	Eye movements were recorded before and after training	Eye movements	Mean horizontal saccadic amplitude, mean fixation duration.	No significant increase of saccade amplitude during the pop-out exploration task in the group of participants who demonstrated an increase of anti-saccade amplitude during the adaptation training (*p =* 0.70). Amplitude of saccades significantly decreased following the delayed shift training (*p =* 0.04) and following the no shift training for the group in which the adaptation training was inefficient (*p =* 0.02). For the treated-side- a significant increase of the amplitude of return leftward saccades for the LHH participants with positive slopes following the adaptation training only (*p =* 0.001). A decrease in the number of leftward saccades in LHH participants following the adaptation training.
Iorizzo et al (2011) [55]	Case-control study	*n =* 12 HH (*n =* 6) Healthy controls (*n =* 6)	Stroke (*n =* 6)	Adults		Eye and head movements were recorded while subjects were asked to visually detect and track flying basketballs as they appeared in the virtual world.	Eye and head movements	Fixation durations and numbers, fixation distribution, degrees of head movements	HH participants made shorter fixations (227 ms ± 19) than controls (316 ms ± 12) when sitting, but not walking (161 ms ± 15; 158 ms ± 10 respectively). When sitting, hemianopic subjects made more fixations (211 ± 97) than controls (89 ± 33) over a 2-min period. When walking, they made a similar number of fixations (184 ± 48) over the first 5 paths as controls (118 ± 35). Fixation bias into the BHF when sitting was greatly exaggerated (4.7 ± 2.1°) relative to that in sitting controls (1.1 ± 0.8; *p =* 0.002). While walking, subjects placed most of their fixations in the side of their BHF (*p =* 0.002). Head movements were generally small, with a standard deviation averaging just above 0.2° in the sitting condition and close to 1° in the walking condition, with no significant differences between controls (0.73 ± 0.17) and hemianopes (0.84 ± 0.29).
Machner et al (2009a) [56]	Case-control study	*n =* 18 HH (*n =* 9) Healthy controls (*n =* 9)	Infarction (*n =* 7) Haemorrhage (*n =* 2)	Adults	N/A	Participants performed an exploratory visual search task and eye movements were recorded.	Eye movements	Number of fixations, Saccades in the BHF (%), amplitude of saccades towards the blind/ intact hemifield, item refixations in the BHF (%)	Significant differences were found on the number of fixations: LHH participants (57 ± 3), RHH participants (104 ± 11) and controls (28 ± 1; *p <* 0.001). In both LHH and RHH participants, the number of saccades did not differ significantly between the blind and the intact hemifield. In LHH participants saccades towards the (blind) left side were slightly but significantly smaller than the rightward saccades (5.8 (±0.5) /6.3 (±0.4), *p <* 0.05). This was true for healthy controls (6.3 (±0.2) /7.0 (±0.3)). RHH participants’ leftward saccades were slightly larger than the rightward saccades, but the difference was not significant. The mean saccadic amplitude differed significantly (*p <* 0.01) between controls (6.5° ± 0.1), LHH (6.0° ± 0.2), and RHH participants (4.6° ± 0.2). Item refixations increased for RHH participants (*d =* 31.3% ± 4.2, *p <* 0.001) and LHH participants (*d =* 17.0% ± 3.9, *p <* 0.001), when compared to controls.
Machner et al (2009b) [57]	Case-control study	*n =* 27 HH (*n =* 9) Simulated HH (*n* = 9) Healthy controls (*n =* 9)	Infarction (*n =* 7) Haemorrhage (*n =* 2)	Adults	N/A	Eye movements were recorded while subjects searched for targets among distractors and indicated target detection by clicks.	Eye movements	Fixation duration, item refixations, number and amplitude of saccades	For participants with simulated HH, mean fixation duration (263 ms ± 6) was longer (*p <* 0.05) than in controls (230 ms ± 6) and HH participants (243 ms ± 6). HH participants made more saccades (89 ± 7) than controls (29 ± 7, *p <* 0.001) and simulated HH participants (43 ± 7, *p <* 0.001). Participants’ mean saccadic amplitude was significantly smaller than in controls (*d =* 1.2° ±0.4, *p <* 0.01) but not than in virtuals (*d =* 0.6° ± 0.4, *p* > 0.05). HH participants showed a higher rate of refixations than controls (*d =* 23.3 ± 3.2, *p <* 0.001). Simulated HH rates of refixations were not just lower than in HH participants (*d =* 41.3 ± 3.2, *p <* 0.001) but also than in the controls (*d =* 18.0 ± 3.2, *p <* 0.001). In both groups, HH participants and simulated HH, there were no significant differences between saccades which landed in the left or right half of the screen concerning total number, amplitude or fixation duration. No significant differences were found when comparing rates of item refixations between the two absolute hemifields. For all three groups, numbers of saccades towards either side were evenly distributed.
Mannan et al (2010) [58]	Before and after study	*n =* 31 HH (*n =* 18, 13 left, 13 right)	Infarction (*n =* 22) AVM (*n =* 6), Temporal lobectomy (*n =* 1), Glioma (*n =* 1), Cystic teratoma (*n =* 1)	Adults	Visual search training. 40-min sessions, 5x per week totalling 20 sessions.	Participants searched for randomly positioned target presented for 3 s (line, square or triangle) among 40 distractors	Eye movements	Proportion of initial saccades made into the BHF, mean duration for fixations made in BHF only, mean duration for fixations made in SHF only	Participants made exactly the same proportion initial saccades (50%) before and after training. BHF: 54 (3), 51 (2), 51 (3), 56 (3) (visit 1,2,3,4 respectively). SHF: 49 (3), 48 (2), 48 (3), 52 (3) (visit 1,2,3,4 respectively). The proportion of fixations made in hemianopic hemispace also remained unchanged after training (47%). Participants made a significantly greater proportion of fixations in hemianopic hemispace after training when the target was located on this side. Participants made significantly fewer fixations compared to both visits 1 and 2. Mean durations of fixations made in the BHF did not change after training (194 (13), 197 (11), 189 (10), 188 (7) ms). Participants made a significantly larger initial saccade after training (visit 2: 6.27°, visit 3: 6.81°). The mean amplitude of saccades following the initial saccade showed no effect of training. Participants made 57% of their saccades in the direction of the BHF if the target was in hemianopic hemispace, while only 25% saccades were in the BHF for targets in intact hemispace.
Martin et al (2007) [26]	Case-control study	*n =* 8 HH (*n =* 4) Healthy controls (*n =* 4)	Stroke	Adults	N/A	Participants’ gaze patterns were analysed while they assembled wooden models.	Eye movements	Saccade duration, saccade amplitude, saccade peak velocity, fixation duration, accuracy of fixations, look-ahead and look-back fixations	HH participants made slightly longer saccades (73 ± 1.5 ms) compared to controls (69 ± 2.5 ms). The difference was not statistically significant (*p =* 0.34). HH participants made slightly larger saccades (9.32° ± 0.9), than controls (8.29° ± 0.5). Difference was not statistically significant (*p =* 0.33). Saccade peak velocity was slightly higher in HH (163.2 ± 11.5d/s) than controls (155.3 ± 9.9d/s). Again difference did not reach statistical significance (*p =* 0.63). HH participants exhibited slightly shorter fixation durations (510 ± 151 ms) than controls (561 ± 41 ms; *p =* 0.72). Fixation durations did not differ significantly between the sighted and blind hemifields of each hemianope. Hemianopes had an average of 5.0 ± 4.0 look-back fixations, while controls had an average of 1.5 ± 1.73 (not statistically significant).
Pambakian et al (2000) [25]	Case-control study	*n =* 16 HH (*n =* 8, 4 right, 4 left) Healthy controls (*n =* 8)	Infarct (*n =* 4) AVM (*n =* 2) Cystic suprasellar teratoma (*n =* 1) Meningioma (*n =* 1)	Adults	N/A	Eye movements were recorded while participants viewed 22 images of real scenes. Subjects viewed each image for 3 s, initially in a spatially filtered form in which much of the semantic content had been removed, and then in their unfiltered, original form.	Eye movements	Number and duration of fixations, mean amplitude of first saccade, mean amplitude of all measured saccades, scanpath length, proportion of fixations made in BHF, percentage of saccades made towards the BHF	Participants made significantly more fixations than control subjects while viewing filtered (8.7 (0.2), 7.7 (0.1) respectively, *p <* 0.0002) but not unfiltered pictures (9.8 (0.1), 9.6 (0.1), *p <* 0.2). Participants’ fixations duration were significantly shorter than those of control subjects (312 ms (8), 372 (11), *p <* 0.0001 for filtered images) and (286 ms (6), 302 (11), *p <* 0.2 for unfiltered images). The mean amplitude of the first saccade was significantly larger for participants than control subjects for both filtered (3.2° (0.1), 2.3° (0.1), *p <* 0.0001) and unfiltered images (3.3° (0.1), 2.6° (0.1)). Mean amplitude of the remainder of the saccades did not reach significance. Participants produced significantly longer scanpaths than controls while viewing both filtered (32.2° (1.4), 26.8° (0.8), *p <* 0.0415) and unfiltered images (39.8° (1.0), 37.2° (0.8), *p <* 0.0415). Viewing filtered images, left and right hemianopes made significantly more fixations into the BHF (LHH: 51 (1.4), controls (42 (1.2), *p <* 0.05); RHH: 63 (1.6), controls (58 (1.2), *p <* 0.05). HH participants spent significantly longer making fixations in their respective BHF compared to controls for both filtered (LHH: 71 (2.3)/42 (1.9), *p <* 0.05; RHH: 71 (2.5) /58 (1.9), *p <* 0.05) and unfiltered images (LHH: 59 (2.9)/50 (2.1), *p <* 0.05; RHH: 58 (2.3)/50 (2.1), *p <* 0.05). Participants made significantly more saccades towards their blind side compared to controls for filtered (LHH:71 (2.1)/44 (2.5), *p <* 0.05; RHH:71 (2.4)/56 (2.5) *p <* 0.05) and unfiltered images (LHH:61 (2.6)/50 (2.1); RHH:60 (2.1)/50 (2.1), *p <* 0.05).
Papageorgiou et al (2012) [59]	Case-control study	*n =* 60 HH (*n =* 20) Quadrantanopia (*n =* 10) Normal subjects (30)	Vascular brain lesion	Adults	N/A	Participants performed a collision avoidance task with moving objects at an intersection under two difficulty levels. Based on their performance (i.e. the number of collisions), participants were assigned to either HP or LP subgroups by the median split method.	Eye movements	Saccade amplitudes, scanpath length, mean number of gaze shifts, number and the duration of fixations, mean gaze eccentricity	Compared to LP participants, visual exploration of HP participants at both traffic densities was characterised by larger saccadic amplitudes towards the BHF (18.06° HP, 14.58° LP for 50% density, *p <* 0.01). Shorter saccades to the BHF were evident for HP (18.06°), LP participants (14.58°) compared to controls (22.03°). HP participants showed significantly longer scanpaths than LP participants (1126.21° and 793.38°, *p <* 0.001 at 75% density). HP participants showed more gaze shifts (10.5) and larger mean gaze eccentricity (22.38°) compared to LP (7.16, 17.22° respectively). No significant differences between HP and LP participants regarding total fixation number, fixation duration, proportion of fixations and proportion of gaze eccentricity to the BHF. Both HP and LP participants showed increased numbers of fixations to the BHF compared to their SHF (59.88/40.12; 59.52/40.48 respectively).
Passamonti et al (2009) [60]	Controlled before and after study	*n =* 24 HH (*n =* 12, 6 right, 6 left) Healthy controls (*n =* 12)	Vascular (*n =* 7) Trauma (*n =* 3) AVM (*n =* 1) Craniotomy (*n =* 1)	Adults	Audio-visual (experimental) training vs visual (control) training. 4 h over a period of 2 weeks	Oculomotor responses during a visual search task and a reading task were studied before and after training	Eye movements	Number and duration of fixations, refixations, saccadic amplitude and duration, length of scanpath, number of progressive and regressive saccades, return sweep	Participants took more time than control subjects to explore the visual display, and also produced more fixations and refixations, resulting in a longer scanpath. Saccades were slower and shorter in amplitude (*p <* 0.05 in all comparisons). After the AV training—significantly fewer fixations (LH: 95 (24) at S1 and 60 (16) at S4, RH: 100 (32) at S1 and 83 (29) at S4). Saccadic duration was markedly reduced (130 ms (30) at S1, 84 ms (15) at S4). Mean saccadic amplitude significantly increased (LH:7.69 (0.7) at S1; 9.32 (0.2) at S4). Length of scanpath significantly reduced in RHH and LHH participants after training LHH:624 (90) at S1;456 (72) at S4, RHH:746 (165) at S1;587 (198) at S4. Rate of refixations (%) significantly reduced (RHH: 11 (2)–5 (2) and LHH: 12 (3)–4 (2)).
Reinhard et al (2014) [61]	Case-control study	*n =* 47 HH (*n =* 33, 15 right, 18 left) Healthy controls (14)	Infarction (*n =* 12) Traumatic (*n =* 6) Haemorrhage (*n =* 4) Postoperative complications (*n =* 7) AVM (*n =* 2) Unknown (*n =* 2)	Adults	N/A	Eye movements were recorded during saccadic and fixation tasks	Eye movements	Landing accuracy of saccades, Fixation stability (FS) after landing, distribution of fixational eye movements (FEMs)	The mean number of hypermetric saccades during gaze shift to the left was approximately 2.5 times higher in LHH (1.2 ± 0.2 SEM) compared with RHH (0.5 ± 0.1 SEM). The mean number of hypermetric saccades was much higher during gaze shifts to the BHF (1.2 ± 0.2 SEM) than to the SHF (0.5 ± 0.2 SEM) (*p =* 0.003) and compared with control subjects (0.26 ± 0.08 SEM) (*p =* 0.0001).
									Fixation stability after landing was lower after saccades to the BHF compared with the seeing side with the mean FS of 67.5% vs 74.5%, respectively. Mean FS after gaze shifts to the seeing side was significantly higher than the FS after gaze shifts to the BHF (*p =* 0.013). Fixational eye movements were asymmetric towards the hemianopic side. In participants with LHH, the mean was shifted to -1.312° (left) (*p <* 0.0001). In participants with RHH, the mean was shifted to 2.6° (right) (*p* < 0.0001).
Roth et al (2009) [62]	RCT	*n =* 30 HH	Ischemic stroke (*n =* 22) Haemorrhage (*n =* 4) AVM (*n =* 1) Arachnoidal cyst (*n =* 1) Cerebral abscess (*n =* 1) Head injury (*n =* 1)	Adults	Participants were randomly assigned to distinct groups performing for 6 weeks either Explorative Saccade Training (EST) (a digit-search task) or Flicker Training (FT) (BHF stimulation by flickering letters). Training sessions lasted 30 min each.	Eye movements during initial fixation and subsequent free exploration of natural scenes were recorded	Eye movements	Fixation number, fixation stability	Only with EST did the number of fixations during natural scene exploration increase towards the BHF (follow-up/pre difference, 238%). The EST group showed decreased fixation stability (post/pre, 43%) and increased asymmetry of fixations towards the BHF (post/pre, 482%).
Schoepf et al (1996) [63]	Case-control study	*n =* 22 HH (*n =* 12, 5 right, 3 left) Healthy controls (10)	Infarction (*n =* 6) Stenosis (*n =* 1), Neuritis (*n =* 1)	Adults	N/A	Eye movements were recorded while subjects participated in two experiments: reading experiment and predictable target experiment	Eye movements	Accuracy and distribution of eye-head gaze saccades at different target frequencies, mean velocity of eye-head gaze saccades, mean amplitude of eye-head gaze saccades	Participants with RHH demonstrated a comparable distribution of correct saccades in both hemifields (M = 60.30 ± 3.2). In LHH participants, the percentage of undershooting and correct saccades was nearly the same and stayed around 40% in the BHF (median 38.5%, range 35.9% to 41.1%). In the better adapted RHH participants, amplitudes of eye-head gaze saccades became significantly increased and corresponding velocities faster with increasing target frequencies. No significant differences between saccades directed either into the SHF or into the BHF.
Tant et al (2002) [64]	Case-control study	*n =* 45 HH (*n =* 29, 15 right, 14 left) Healthy controls (*n =* 16)	Stroke (*n =* 23) Tumour (*n =* 3) Hydrocephalus (*n =* 1) Head injury (*n =* 2)	Adults	N/A	Hemianopic condition was simulated in healthy subjects and their scanning performance on a dot counting task was compared to their own “normal” condition and to real HH participants’ performance	Eye movements	Number and duration of the fixations, number and amplitude of the saccades, length of the scanpath	HH participants displayed significantly higher number of fixations (33) compared to simulated HH (24) and normal controls (14). Simulated HH provoked higher duration of fixations (560 ms) compared to HH participants (380 ms) and normal controls (390 ms). In both HH groups, there were more fixations in ipsilateral (sHH 12.5; HH 15.7) than in contralateral hemispace (sHH 8.9, HH 9.1 ) Ipsilateral saccadic amplitudes were smaller than contralateral amplitudes in simulated (9.08°, 10.81°) and true HH (7.00°, 8.2°). The amplitudes of saccades into the BHF were smaller than into the SHF, for both left- and right-sided sHH.
Turton et al (2018) [65]	Cohort study	*n =* 9HH (*n =* 7, 1 right, 6 left) Quadrantanopia (*n =* 1) Partial visual field loss with macular involvement (*n =* 1)	Ischemic stroke (*n =* 6) Haemorrhage (*n =* 3)	Adults	Participants received an intensive course of scanning and search training using various process activities and occupations. Training lasted 1 h, three times a week for 3 weeks		Head movement	Head yaw and pitch	Increased searching on blind side, from head movement, following intervention (for *n =* 3 of 9)
Wood et al (2011) [66]	Case-control study	*n =* 60 HH (*n =* 22, 5 right, 17 left) Quadrantanopia (*n =* 8) Healthy controls (*n =* 30)	Cerebrovascular accident (*n =* 12) Tumour (*n =* 2) AVM (*n =* 2) Congenital brain abnormality (*n =* 1) Trauma (*n =* 5)	Adults	N/A	The driving performance of each participant was assessed under in-traffic conditions along 6.3 miles of non-interstate driving in residential and commercial areas of a city. Eye and head movements and lane keeping were rated.	Eye, head and shoulder movements	Scoring of head movements, the extent of eye movements	HH participants exhibited more head movements (57.81) than quadrantanopes (51.06) and normal controls (50.17). They also had more shoulder movements (5.45) than quadrantanopes (4.00) and controls (3.37). HH and quadrantanopic participants made significantly more head movements into their BHF than SHF. 59% of all head movements were made into the BHF compared with the SHF (33.18 vs. 22.66), with 30% more large head movements made into the BHF compared with the SHF (13.67 vs. 10.53) and 60% more small head movements made into the BHF relative to the SHF (19.51 vs.12.13). The safe drivers made 70% more small head movements into their blind than seeing field (21.13 vs.12.30), whereas the unsafe drivers made only 20% more small head movements into their BHF than SHF.
Zangemeister et al (1982) [67]	Case series	*n =* 6 HH (4 right, 2 left)	Haemorrhage (*n =* 2) Infarction (*n =* 1) AVM (*n =* 1)Occipital porencephaly (*n =* 1) Transsynaptic retrograde degeneration (*n =* 1).	Adults	N/A	Eye-head movements were recorded while participants searched for target presented in the horizontal plane within 10° of either side of centre.	Eye and head movements	Latencies of eye (EL) and head (HL) movements, head movement velocities and compensatory eye movement (CEM) velocities.	Head movements to the SHF followed eye movements with the same 50 ms delay observed in normal subjects, but there was an additional delay of 80 ms on the average in head movements to the BHF. Eye movements were symmetrically delayed by about 110 ms more than in normal subjects. In participants with acquired HH, the ratio of CEM velocity to head velocity (CEM gain) was small with head movements towards the SHF, great with movements towards the BHF. Head movements towards the BHF often showed a staircase pattern. Head movements into the SHF often consisted of a single, large movement.
Zihl et al (1995) [23]	Case-control study/ before-after	*n =* 74 HH (*n =* 60, 30 left, 30 right) Healthy controls (*n =* 14)	Cerebrovascular (*n =* 55) Traumatic (*n =* 5)	Adults	A subgroup of participants (*n =* 14) were systematically treated with saccadic eye movement training to compensate better for their hemianopic field loss. 30 min each session (total of 16 sessions on average)	Eye movements were recorded while subjects inspected a pattern of 20 white dots randomly scattered over the screen, with a mean spatial separation of 7°.	Eye movements	Number and duration of fixations, amplitude of saccades, rates of repetitions of fixations, length of scanpath	The scanning pattern of “pathologic” HH characterised by significantly higher numbers of fixations not only in the BHF, but also in the SHF (38/27 in LHH and 45/24 in RHH) compared to controls (09/11). The repetition rates of scanpath and fixations were significantly higher in both hemifields (37/45 in LHH and 26/48 in RHH) compared to controls (02/13). Amplitudes of saccadic eye movements and durations of fixations did not differ significantly between the groups. Length of scanpath significantly increased in HH participants (LHH: SHF/BHF:100.7/159°; RHH: SHF/BHF 84.3/180°) compared to healthy controls (36.9/49°). The repetition rates of scanpaths and fixations parameters significantly reduced after treatment to less than one half as compared to pre-treatment (scanpath: 17% and 8%; fixations: 44% and 22%, *p <* 0.0001). Mean amplitudes of saccades were significantly larger after practice (mean increase was 0.9°), while mean durations of fixations remained unchanged.
Zihl et al (1999) [68]	Case-control study	*n =* 100 VFD (*n =* 70: 40 HH, 20 quadrantanopia, 10 HH). Normal controls (*n* = 30)	Stroke	Adults	N/A	Eye movements were recorded during the inspection of a stimulus pattern. The stimulus array consisted of 20 dots.	Eye movements	Number and duration of fixations, fixation repetition, saccadic amplitude, scan path length	In the impaired groups, scanning times on average were about twice as long and scan paths were twice as large. The number of fixations exceeded that of the unimpaired group by 55–71% (M 75.9%); the rate of refixations exceeded the corresponding values by 2–28% (M 18.9%). Mean fixation durations were sometimes shorter (0.26 s (0.04)) and sometimes longer (0.32 s (0.08)) than controls (0.28 s (0.05)). Subjects had smaller saccadic amplitudes (5.0° (0.8) than controls (5.4° (0.7). Subjects showed larger scan paths (295.9° (51.4)) compared to controls (137.6° (46.5)).

*AS* anti-saccades, *AV* audio-visual exploration training, *AVM* arteriovenous malformation, *BHF* blind hemifield, *CEMs* compensatory eye movements, *CFD* cumulative fixation duration, *CVS* comparative visual search, *d/s* degree per second, *d* difference, *DC* dot counting, *EL* latencies of eye movement, *EST* explorative saccade training, *FEMs* fixational eye movements, *FS* fixation stability, *FT* flicker training, *GF* glance frequency, *GPP* glance proportion in percentage, *HGA* horizontal gaze activity, *HGD* horizontal gaze distribution, *HH* homonymous hemianopia, *HHf* homonymous hemianopia who failed, *HHp* homonymous hemianopia who passed, *HL* latencies of head movement, *HP* high performance, *IQR* interquartile range, *LHH* left homonymous hemianopia, *LP* low performance, *M* mean, *Pf* participants who failed, *PGP* glance proportion in percentage, *Pp* participants who passed, *RHH* right homonymous hemianopia, *SD* standard deviation, *SEM* standard error of the mean, *SHF* sighted hemifield, *sHH* simulated homonymous hemianopia, *VFD* visual field defect, *VT* visual exploratory training

Eye movements were recorded using an infrared eye tracking in 16 studies [26, 43, 44, 46, 47, 49–52, 54, 55, 59–62, 64]. In two studies, subjects’ eye movements were recorded using a scleral search coil system [56, 57]. Other methods used to record eye movements were as follows: infrared reflection oculography [48, 67], electro-oculography [53], pupil-corneal-reflection method [23, 68], digital, head-worn video camera [63, 66] and the P scan system [25, 58]. In all the included studies, gaze parameters were measured while head movements were unrestricted.

Head movements were recorded in eight studies by means of a head-mounted binocular infrared video pupil tracker [44, 51, 55], a high-resolution accelerometer [63], digital head-worn video camera [65, 66], a low-torque potentiometer [67] or a remote infrared system [45]. Shoulder movements were recorded in two studies by means of a video camera [51, 66].

#### Eye movement parameters

##### Number, duration of fixations and percentage of fixation repetitions

The most commonly reported eye movement parameters included number of fixations, duration of fixations and percentage of fixation repetitions, measured in 17 studies (*n =* 524). Participants in these studies were tested in various visual tasks, from those requiring mostly simple visual scanning (e.g. dot counting) to more cognitively demanding visual search tasks (e.g. comparative visual search task).

In comparison to normal controls, the scanning pattern of participants with HH was characterised by significantly higher numbers of fixations, a higher proportion of fixations towards the BHF and a higher proportion of refixations [23, 25, 57].

Different studies showed different findings regarding mean fixation durations. Eye movement recording during the inspection of a stimulus pattern showed that mean fixation durations were longer in the impaired groups compared to controls [68]. However, compensatory behaviour of participants with HH while they assembled models and viewing naturalistic pictures was characterised by slightly shorter fixation durations than controls. Fixation durations did not differ significantly between the SHF and BHF while participants assembled wooden models [26]. Participants made significantly more fixations in the area corresponding to their respective BHF compared to controls while viewing filtered images [25].

In the naturalistic setting of a driving simulation, no significant difference was found regarding the duration of fixations between the healthy controls and participants with HH [44].

When participants with HH were divided into two subgroups by the median of their task performance into “high performance” and a “low performance” groups, participants in the high performance group showed no statistically significant differences from the normal controls regarding fixation number, duration of fixations and proportion of refixations [44, 50, 59].

Practice-related improvement in scanning efficiency was reported in five studies (*n =* 169) [23, 49, 58, 60, 62]. The number of fixations and refixations significantly reduced after treatment by at least 20% as compared to pre-treatment values. After explorative saccade training [62], the number of fixations during natural scene exploration increased significantly towards the BHF (follow-up/predifference, 238%). However, the proportion of fixations made into the BHF did not change as a result of visual search training [58].

##### Number and the duration of saccades

The number and duration of saccades were measured in five studies (*n =* 111). In a driving simulation experiment and visual search tasks, no statistically significant differences were found between participants with HH and healthy controls concerning the number and duration of saccades [26, 44, 57]. Passamonti et al. studied oculomotor responses during a visual search task before and after visual (control) or audio-visual (experimental) training [60]. Before training, duration of saccades was significantly longer in participants with HH compared to controls. After training, saccadic duration markedly reduced by about 40% in participants with HH. Keller et al. also reported that audio-visual stimulation training (AVT) significantly increased the number of saccades in participants with HH compared to visual stimulation training (VT) [53]. Participants in the AVT group nearly doubled the number of saccades into their BHF while participants in the VT group increased the number of saccades by only about 11%.

##### Amplitude, peak velocity of saccades and scan path length

The amplitude of saccades was measured in 14 studies (*n =* 497) [23, 25, 26, 44, 47, 48, 50, 53, 54, 57, 59, 60, 64, 68] while the peak velocity of saccades was measured in two studies (*n =* 49) [26, 44]. Scan path length (the sum of all saccadic amplitudes) was measured in four studies (*n =* 124) [25, 50, 59, 60].

Compared to normal controls, visual exploration by participants with HH was mostly characterised by significantly smaller mean saccadic amplitude. In contrast, analysing the gaze patterns of participants with HH and visually intact controls while they assembled wooden models [26] and during memory recall in an imagery task, a non-imagery task (verbal fluency) and a visually guided task [47], participants performed a roughly similar number of saccades of similar amplitude compared to normal controls.

Recording eye movements in a driving simulation experiment [44], participants with HH had significantly lower peak velocity of saccades compared to normal controls.

Participants with HH whose performance on the relative search task was rated as high showed no statistically significant differences from the normal controls regarding the amplitude, peak velocity of saccades and scan path length. The low performance group showed significantly smaller amplitude, lower velocity and longer scanpaths than either the control or high performance groups [44, 50, 59].

Saccadic training which took different forms across different studies [23, 53, 54, 60] demonstrated increased saccadic amplitude and reduced length of scan path compared to pre-training. Scan path length significantly reduced after audio-visual training by about 50% compared to pre-training.

##### Percentage of saccades made towards the blind hemifield

Four studies (*n =* 92) measured the proportion of saccades made towards the BHF [25, 56–58]. Recording eye movements of participants with acute and chronic HH while they performed an exploratory visual search task, Machner et al. found that the number of saccades did not differ significantly between the blind and the intact hemifield [56, 57]. However, Pambakian et al. examined the scanpaths of participants with HH while viewing naturalistic pictures in their original and also spatially filtered forms and participants made significantly higher proportion of saccades towards their BHF compared to the SHF for both filtered and unfiltered images [25]. This variation supports the view that the contralesional bias towards the BHF reflects a compensatory eye movement strategy which may be due to an attentional shift developed over time.

After visual search training, participants with HH made a higher proportion of saccades in the direction of the target, i.e. participants made more contralesional (57%) than ipsilesional saccades (23%) when the target was in hemianopic hemispace. After training, participants were able to locate targets within a larger area of their blindfield within a single saccade [58].

##### Gaze eccentricity on the blind and seeing sides

Two studies (*n =* 72) measured gaze eccentricity (i.e. the average gaze position from the straight-ahead position) on the blind and seeing hemifields [43, 59]. In a driving simulator experiment, Alberti et al. calculated gaze and pedestrian eccentricities with respect to the car heading direction for all pedestrians that appeared at approximately 14° on the blind side. It was reported that only 40% of scans to reach the pedestrian on the BHF were made within 1 s of the pedestrian appearing compared with approximately 70% on the SHF [43]. Papageorgiou et al. also examined gaze patterns applied by participants with HH under virtual reality conditions in a dynamic collision avoidance task. Low-performing participants with HH exhibited lower mean gaze eccentricity than controls and showed a higher proportion of gaze eccentricity to the BHF at lower traffic densities [59]. High-performing participants with HH, however, had higher mean gaze eccentricity than controls and higher proportion of gaze eccentricity to the BHF at 50% density compared to 75% density [59].

##### Horizontal gaze activity

Two studies (*n =* 60) investigated the horizontal gaze exploration ability of participants with HH by assessing the horizontal standard deviation of the pupil (measured on the *x*-axis) [51, 52]. Kasneci et al. recorded eye movements of participants with HH while they collected products placed on two supermarket shelves. No statistically significant difference was found regarding the horizontal gaze activity between participants with HH and age-matched controls [52]. Similarly, a study that assessed the on-road driving performance of HH participants with simultaneous eye and head tracking found no statistically significant difference in the horizontal gaze activity between participants with HH and healthy controls [51].

##### Glance proportion and glance frequency

Two studies (*n =* 60) measured the proportion of gazes towards a defined area of interest during a specific time interval and glance frequency. In a supermarket search task, no significant difference was found between the participant subgroups regarding the proportion of glances beyond the 30° visual field [52]. In a study to assess on-road driving performance, participants with HH who passed the driving test glanced more towards the BHF than participants who failed [51].

##### Mean amplitude and velocity of eye-head gaze saccades

One study (*n =* 22) measured the amplitude and velocity of eye-head gaze saccades (saccades accompanied by head movements) [63]. According to their adaptive state of reading, the better adapted hemianopic participants showed significantly increased amplitudes of eye-head gaze saccades and corresponding velocities were faster with increasing target frequencies.

No significant differences were reported between saccades directed either into the SHF or into the BHF. Less adapted participants, however, showed significantly lower amplitude and velocity of eye-head saccades directed into the BHF than saccades that were aimed into the SHF.

#### Other less reported eye movement parameters

##### Mean deviation of saccade from horizontal and amplitude of first saccade

One study recorded the mean amplitude of all measured saccades and amplitude of first saccade (*n =* 16). This study examined the scan paths of participants with HH while viewing naturalistic pictures in their original and also spatially filtered forms [25]. The mean angle of deviation for participants with HH was significantly lower compared to the control group when viewing filtered and unfiltered images. The mean amplitude of the first saccade was significantly larger for participants with HH than controls for both filtered and unfiltered images.

##### Spatial consistency between gaze positions and spatial distribution of the gaze throughout the task

One study (*n =* 19) recorded the gaze position and its spatial distribution during memory recall of French towns in an imagery task, a non-imagery task (verbal fluency) and a visually guided task in participants with left or right HH [47]. Gaze was constantly shifted across all tasks with respect to their body midline, contralesionally for all participants with HH without neglect and ipsilesionally for the two participants with HH with neglect. For each participant, horizontal shift was statistically significant when compared to healthy controls who systematically positioned their gaze at their body midline when they started their mental imagery.

##### Mean number of gaze shifts

One study (*n =* 60) recorded the mean number of gaze shifts (i.e. gaze transitions between left and right hemifield) in participants with HH under virtual reality conditions in a dynamic collision avoidance task [59]. The subgroup of participants who adapted successfully to their VFD showed increased number of gaze shifts at 50% and 70% traffic density conditions compared to healthy controls. Participants who failed to adapt exhibited lower number of gaze shifts at 50% and 70% traffic density conditions than controls.

##### Progressive and regressive fixations

One study (*n =* 8) recorded the proportion of progressive and regressive fixations in participants with HH and healthy controls while they assembled wooden models [26]. Progressive fixations were defined as fixations upon a location in the 10 s prior to a pickup from that location, while regressive fixations were defined as fixations to a location within 10 s after a pickup from that location, with at least one fixation to a different location since the pickup. Participants with HH exhibited a significantly higher proportion of progressive fixations than controls. Regressive fixations were very rare [26].

##### Number of progressive and regressive saccades

One study (*n =* 24) recorded the number of progressive (left-to-right eye movement between fixations) and regressive (right-to-left eye movements) saccades. Passamonti et al. recorded oculomotor responses of participants with HH during a visual search task before and after AVT. Before training, HH participants had significantly higher number of progressive and regressive saccades compared to controls. After AVT, participants with RHH made fewer number of progressive saccades and fewer mean number of regressions compared to pre-training. Participants with LHH made slightly fewer progressive and regressive saccades compared to pre-training [60].

##### Landing accuracy of saccades and fixation stability after landing

Landing accuracy of saccades and fixation stability were measured in one study (*n =* 47) aimed to detect potential spontaneous adaptive mechanisms in participants with HH during saccadic and fixation tasks [61]. Landing accuracy was decreased in participants with HH, indicated by significantly more hypometric (single-step saccades in which the eye undershoots the target) and hypermetric saccades (saccades that overshoot the target) to the BHF compared with the SHF. Fixation after landing in participants with HH was less stable on the BHF compared with the SHF.

#### Head movements

Head movements were recorded in eight studies (*n =* 221) [44, 45, 51, 55, 63, 65–67]. Examining the head movement parameters during various visual tasks (e.g. visual search during driving and tracking flying basketballs), no statistically significant differences regarding the number, the amplitude, the duration and the peak velocity of head movements were reported between participants with HH and healthy controls [44, 55]. Bowers et al. [45] examined the effects of HH on head scanning behaviours using a driving simulator. Drivers with HH were found to make a greater proportion of head scans overall to the blind side when compared to normal control drivers. However, amplitudes of scans were smaller for HH than for control drivers. For participants with HH, BHF scans were not larger than SHF scans. Similarly, head scanning behaviour of participants with HH during on-road driving was characterised by increased head movements in the direction of the BHF, particularly for those with a left-sided defect [51]. On average, 59% of head movements were made into the BHF compared with the SHF, with large (+ 30%) and small (+ 60%) head movements made into the BHF significantly greater than into the SHF [66].

In a study that examined eye-head coordination in HH, participants seemingly simplified search and fixation strategies by minimising or eliminating head movements and relying solely on eye movements instead with significantly greater delays in head movements towards the BHF [67]. Similarly, Schoepf et al. [63] examined the influence of target predictability on the distribution of coordinated eye-head gaze saccades in participants with HH. The head contribution to the gaze shift was often reduced with significantly longer delays in head movements towards the BHF.

For rehabilitation purposes, Turton et al. [65] assessed the acceptability of search training for people with visual field loss after stroke and reported increased searching on the blind side, from head movement, following the intervention.

The variation in the degree of head movement employed as a compensatory strategy by participants with HH across studies could be attributed to how well they adapt to their visual field loss; better adapted patients seem to use more head movements towards their BHF compared to patients who fail to adapt.

#### Shoulder movements

Shoulder movements were recorded in two studies (*n =* 80) [51, 66]. Scanning behaviour of participants with HH during on-road driving [66] was characterised by a statistically significant increase in shoulder movement compared to controls. Participants with HH who failed the driving assessment displayed significantly less shoulder movements than control subjects and participants with HH who passed the driving test [51].

##### Summary of key findings regarding movement parameters in true hemianopia

The gaze behaviour of participants with HH differs from that of visually intact observers when performing simple laboratory as well as naturalistic tasks. Participants with HH showed significantly increased number of fixations, proportion of fixations to the BHF, scan path length and repetition of fixations. Visual exploration by participants with HH was mostly characterised by a significantly increased number and duration of saccades and scan path length with shorter mean saccadic amplitude and lower peak velocity of saccades. In contrast to the scanning behaviour of participants under simulated HH, participants with HH made significantly more hypometric and hypermetric saccades to the BHF than saccades made into the SHF. Participants with HH also displayed significantly lower amplitude and velocity of eye-head saccades directed into the BHF than saccades that were aimed into the SHF.

The absence of a compensatory gaze bias towards the BHF or of compensatory saccadic search strategies by participants with post-stroke HH was evident during simple, naturalistic tasks (e.g. assembling wooden models). This may reflect the static nature of the task environment which eliminated the need for participants to perform new visual searches and lent strength to the hypothesis that participants with HH might indeed place greater reliance on spatial memory when performing simple, real-world tasks. Similarly, there was no direction-specific bias in participants with acute HH during an exploratory visual search task, i.e. saccades directed towards the BHF did not differ concerning frequency and amplitude from those directed towards the SHF. This suggests that probably due to their acute stage of stroke a compensation has not taken place yet. No significant differences between participants with HH and healthy controls regarding the number, the amplitude, the duration and the peak velocity of head movements were found during various visual tasks. Participants’ head scanning behaviour during on-road driving, however, was characterised by increased head and shoulder movements in the direction of the BHF compared to healthy controls.

Several groups have trained participants with HH in visual search tasks and have concluded that they adopt compensatory eye and head movement strategies with training. Training confers several advantages on the observer. By making more saccades towards their blind side, they bring ever increasing areas into their seeing side, which they examine with numerous rapid fixations.

## Discussion

The aim of this systematic review was to gather and summarise the available evidence on biomechanical alterations to post-stroke VFD. In simulated HH, healthy adults temporarily deprived of information from half of their visual field tended to preferentially move their eyes towards their sighted field of vision, especially during tasks that required complex cognitive processing [37, 38]. Nonetheless, the bias to preferentially search the SHF persisted, even in easy search, and participants continued to direct eye movements into the SHF even when these eye movements gained them very little new information and impeded search performance. Training or repeated exposure to a simulated VFD led to the development of a more efficient visual search strategy. Participants’ fixations moved progressively deeper into the BHF, and this shift was seen in the easy search condition, and to a lesser extent, in the hard condition [38]. Saccades into the BHF were strongly associated with improved search performance.

The scanning behaviour of participants with true HH was mostly characterised by a significantly increased number and duration of fixations, duration of saccades and scan path length with shorter mean saccadic amplitude and lower peak velocity of saccades [25, 26, 56, 57, 59]. This abnormal scanning behaviour was associated with impaired visual exploration, longer visual search times, target omissions and longer, unsystematic scanpaths. Driving was also considered to be problematic for participants with HH. The majority of on-road studies and simulator experiments highlighted poor steering control, incorrect lane position and difficulty in gap judgment [51, 59, 66]. The gaze pattern of drivers with HH was also characterised by increased numbers of fixations, longer search times, longer saccadic amplitudes and more head movements, particularly towards moving objects of interest on their BHF.

In contrast to the shifting bias observed in simulated HH, participants with post-stroke HH tended to spend more time overall looking to the side associated with the deficit during free viewing tasks. These compensatory strategies of biasing gaze in the direction of the BHF were found to be most evident when participants with HH were in dynamic and unpredictable environments [66], where they could not rely on spatial memory to locate salient objects. The differences found between the simulated HH and the true HH may be attributed to several reasons. First, it may be that because of the limited time duration of simulated HH participants did not develop a consistent search strategy. Second, participants with simulated HH are normally aware that their deficit will end with the experiment, whereas participants with HH would be more motivated and have more time to adapt to a long-term deficit. Third, there may be a particular effect of brain damage over and above the visual deficit that is responsible for the specific eye movement pattern. Tant et al. [64] stated that the visual deficit is the main but not the only factor that contributes to the abnormal oculomotor behaviour seen in post-stroke HH. Fourth, participants with HH move more to their BHF because they possess some residual visual abilities in their affected visual field that guides their search more effectively while healthy participants with simulated HH do not have any residual visual information in their blind side. This hemifield bias could also be related to the fact that eye movements in studies involving participants with HH were largely recorded in the chronic stage [57]. Machner et al. [56] recorded eye movements while participants with HH in an acute stage searched for targets among distractors. Both participants with true and simulated HH displayed no direction-specific bias, i.e. saccades directed towards the BHF did not differ in frequency or amplitude from those directed towards the SHF suggesting that compensation had not yet occurred. Some participants discussed in the true hemianopia studies had visual field defects other than hemianopia, for example quadrantanopia and bitemporal visual field loss. However, in the simulated HH group, only the complete right or left hemianopia condition was simulated, and this might account for some differences found in the scanning behaviour across studies. Finally, some individuals with HH are not aware of their visual field loss (especially when combined with spatial neglect) and cannot use voluntary, cognitive control to guide their blind side scanning or have multiple visual impairments that can exacerbate the impact of their hemianopia.

Several limitations to these conclusions should be acknowledged. One critical consideration is that most of the included studies in this review recruited a small number of participants with post-stroke VFD in their study populations (*n* < 30 in 72% of the included studies). Some studies in this review had a mixture of HH aetiologies and did not focus on a specific stroke survivor population. Few studies explored the importance of head movements in compensatory visual search behaviour of participants with HH. Furthermore, the evidence from these studies is inconsistent and it is not clear why some studies demonstrated that successful task performance is related to an increased number of head movements into the BHF while other studies found no difference. The literature on biomechanical changes to post-stroke VFD varies considerably. Significant heterogeneity exists among the included studies with respect to the selection of biomechanical outcome measures. We recommend that researchers should focus on reporting a small, consistent set of key measures of gaze. Research is warranted for the development of a core outcome set of measurements and it is apparent that some gaze parameters (e.g. proportion of saccades made into the BHF, amplitude of saccades and velocity of movements) appear to have more clinical significance than others (e.g. global/local ratio).

The extent to which conclusions drawn from any experiment using simulated HH can be applied to individuals with visual field deficits may be limited. The method employed to simulate HH works by completely removing all the visual information from one half of the display screen in a gaze-contingent manner. This method of “hard edge” hemianopia is only an approximation of what happens in post-stroke VFD and is not entirely consistent with the effects of damage to postgeniculate visual pathways. The loss of vision in the contralateral visual field is sometimes accompanied by residual visual capacity, known as blind sight [69]. Furthermore, the majority of included studies recorded eye and head movements in stroke survivors with HH in the chronic stage and little is known about search behaviour during exploratory visual search tasks within acute phase, in the first few weeks following a stroke.

Findings from this comprehensive review will underpin future clinical research. The review highlights that numerous studies have explored the eye and head movements and scanning behaviour of participants with HH in well-controlled laboratory-based settings but that there has been only limited investigation into what happens under real-world conditions. Future research should aim to establish the extent to which people with HH use effective scanning strategies in real-world situations, whether they are able to adapt their scanning strategies in response to differing task demands, and whether scanning training can be generalised to everyday mobility tasks. The review outlines an extensive list of eye, head and body movement parameters that have been used in the scientific literature to examine scanning behaviour in individuals with HH.

Findings from this review have important implications for clinical practice. Some stroke survivors appear to spontaneously adopt some compensatory strategies [68], and those who do not can be trained to do so. One effective strategy to compensate for a lateralised field deficit is to fixate and saccade as far into the BHF as possible to maximise the proportion of the search area that falls into the SHF [25, 38, 58, 62]. Based on self-report, encouraging stroke survivors with HH to utilise such strategies in real life improves general functioning [58]. Real-life improvements were demonstrated by Bahnemann et al. [44], who compared participants with HH with high and low hazard detection rates in a simulated driving task on a number of eye and head movement measures. Successful performance appeared to be related to compensatory mechanisms of visual exploratory behaviour, namely, an increase in the amplitude and peak velocity of saccades, widening horizontally the distribution of eye movements and a shift of the overall distribution of saccades into the BHF. Similarly, large eye and head movements directed towards the BHF improved search for specific items in a supermarket [52] and collision avoidance [51, 59, 66].

The benefits of rehabilitation for individuals with HH are often perceived as offering only marginal gains. However, we propose that practitioners should be directed to promote active rehabilitation programmes to improve scanning/search performance for individuals with HH. These could employ simple yet effective, user-friendly techniques that can be practised in people’s own homes, causing minimal disruption to their daily lives.

## Conclusion

This systematic review provides a substantial amount of evidence about the inefficient oculomotor scanning behaviour of individuals with HH and healthy participants with simulated HH. Under simulated HH, participants were consistently biased towards the visible part of their visual field whereas the gaze behaviour of participants with HH was biased in the direction of the blind side. With practice, participants with true and simulated HH developed compensatory mechanisms of visual exploratory behaviour, namely, an increase in the amplitude and peak velocity of saccades, widening horizontally the distribution of eye movements and a shift of the overall distribution of fixations and saccades into the blind side. This evidence can be used to underpin the further development, refinement and implementation of visual rehabilitation programmes for hemianopia.

## Supplementary Information

**Additional file 1.**

**Additional file 2.**

**Additional file 3.**

**Additional file 4.**

## Data Availability

All data generated or analysed during this study are included in this published article [and its supplementary information files].

## Abbreviations

AS: Anti-saccades
AV: Audio-visual exploration training
AVM: Arteriovenous malformation
BHF: Blind hemifield
CEMs: Compensatory eye movements
CFD: Cumulative fixation duration
CINAHL: Cumulative Index to Nursing and Allied Health Literature
CVS: Comparative visual search
d/s: Degree per second
d: Difference
DC: Dot counting
EL: Latencies of eye movement
EST: Explorative saccade training
FEMs: Fixational eye movements
FS: Fixation stability
FT: Flicker training
GF: Glance frequency
GPP: Glance proportion in percentage
HGA: Horizontal gaze activity
HGD: Horizontal gaze distribution
HH: Homonymous hemianopia
HHf: Homonymous hemianopia who failed
HHp: Homonymous hemianopia who passed
HL: Latencies of head movement
HP: High performance
IQR: Interquartile range
LHH: Left homonymous hemianopia
LP: Low performance
M: Mean
MEDLINE: Medical Literature Analysis and Retrieval System Online
NV: Normal vision
Pf: Participants who failed
Pp: Participants who passed
PRISMA: Preferred Reporting Items for Systematic Reviews and Meta-Analyses
PsycINFO: Psychological Information Database
RHH: Right homonymous hemianopia
SD: Standard deviation
SEM: Standard error of the mean
SHF: Sighted hemifield
sHH: Simulated homonymous hemianopia
VF: Visual field
VFD: Visual field defect
VT: Visual exploratory training

## References

[1] Stroke Association. UK. State of the nation stroke statistics-May 2020. https://www.stroke.org.uk/resources/state-nation-stroke-statistics. last accessed 12/11/2020

[2] Rowe FJ, Hepworth LR, Howard C, Hanna KL, Cheyne CP, Currie J (2019). High incidence and prevalence of visual problems after acute stroke: an epidemiology study with implications for service delivery. Plos One..

[3] Pezzullo L, Streatfeild J, Simkiss P, Shickle D (2018). The economic impact of sight loss and blindness in the UK adult population. BMC Health Serv Res..

[4] Hepworth LR, Rowe FJ, Walker MF, Rockliffe J, Noonan C, Howard C, et al. Post-stroke visual impairment: a systematic literature review of types and recovery of visual conditions. Ophth Res An Int J. 2016;5(1):1-43. 10.9734/OR/2016/21767.

[5] Han L, Law-Gibson D, Reding MJS (2002). Key neurological impairments influence function-related group outcomes after stroke. Stroke..

[6] Patel AT, Duncan PW, Lai S-M, Studenski SJ (2000). The relation between impairments and functional outcomes post stroke. Arch Phys Med Rehab..

[7] Cassidy TP, Bruce DW, Lewis S, Gray CS (1999). The association of visual field deficits and visuo-spatial neglect in acute right-hemisphere stroke patients. Age Ageing..

[8] Zhang X, Kedar S, Lynn MJ, Newman NJ, Biousse VJ (2006). Homonymous hemianopia in stroke. J Neuro Ophth..

[9] Jones SA, Shinton RA (2006). Improving outcome in stroke patients with visual problems. Age Ageing..

[10] Granger CV, Divan N, Fiedler RC (1995). Functional assessment scales. A study of persons after traumatic brain injury. Arch Phys Med Rehab..

[11] Ramrattan RS, Wolfs RC, Panda-Jonas S, Jonas JB, Bakker D, Pols HA, Hofman A, de Jong PT (2001). Prevalence and causes of visual field loss in the elderly and associations with impairment in daily functioning: the Rotterdam Study. Arch Ophth..

[12] MacIntosh CJ (2003). Review articles-Stroke re-visited: visual problems following stroke and their effect on rehabilitation. Br Orth J..

[13] Hepworth LR, Rowe FJ. Visual impairment following stroke-the impact on quality of life: a systematic review. Ophth Res. An Int J. 2016;5(2):1–15. 10.9734/OR/2016/23272.

[14] Williams DR (1985). Aliasing in human foveal vision. Vision research..

[15] Atchison DA, Smith G. Optics of the human eye. Oxford: Butterworth-Heinemann; 2000.

[16] Kowler E (2011). Eye movements: the past 25 years. Vision Res..

[17] Bahill AT, Adler D, Stark L (1975). Most naturally occurring human saccades have magnitudes of 15 degrees or less. Invest Ophthalmol Vis Sci..

[18] Hollands MA, Patla AE, Vickers JN (2002). “Look where you’re going!”: gaze behaviour associated with maintaining and changing the direction of locomotion. Exp Brain Res..

[19] Martinez-Conde S, Macknik SL, Hubel DH (2004). The role of fixational eye movements in visual perception. Nat Rev Neurosci..

[20] Rucci M, Poletti M (2015). Control and functions of fixational eye movements. Annu Rev Vis Sci..

[21] Niehorster DC, Peli E, Haun A, Li L (2013). Influence of hemianopic visual field loss on visual motor control. Plos One.

[22] Leff AP, Scott SK, Crewes H, Hodgson TL, Cowey A, Howard D, Wise RJS (2000). Impaired reading in patients with right hemianopia. Ann Neurol..

[23] Zihl J (1995). Visual scanning behavior in patients with homonymous hemianopia. Neuropsychol..

[24] Mena-Garcia L, Maldonado-Lopez MJ, Fernandez I, Coco-Martin MB, Finat-Saez J, Martinez-Jimenez JL, Pastor-Jimeno JC, Arenillas JF (2020). Visual processing speed in hemianopia patients secondary to acquired brain injury: a new assessment methodology. J NeuroEngineering Rehabil..

[25] Pambakian ALM, Wooding DS, Patel N, Morland AB, Kennard C, Mannan SK (2000). Scanning the visual world: a study of patients with homonymous hemianopia. J Neurol Neurosurg Psych..

[26] Martin T, Riley ME, Kelly KN, Hayhoe M, Huxlin KR (2007). Visually-guided behavior of homonymous hemianopes in a naturalistic task. Vision Res..

[27] Rowe FJ, Conroy EJ, Bedson E, Cwiklinski E, Drummond A, Garcia-Finana M (2017). A pilot randomized controlled trial comparing effectiveness of prism glasses, visual search training and standard care in hemianopia. Acta Neurol Scand.

[28] Aimola L, Lane AR, Smith DT, Kerkhoff G, Ford GA, Schenk T (2014). Efficacy and feasibility of home-based training for individuals with homonymous visual field defects. Neurorehab Neural Repair..

[29] Hollands KL, Pelton TA, van der Veen S, Alharbi S, Hollands MA (2016). A novel and simple test of gait adaptability predicts gold standard measures of functional mobility in stroke survivors. Gait Posture..

[30] Howard C, Rowe FJ (2018). Adaptation to poststroke visual field loss: a systematic review. Brain Behav.

[31] Moher D, Liberati A, Tetzlaff J, Altman DG, The PG (2009). Preferred Reporting Items for Systematic Reviews and Meta-Analyses: the PRISMA statement. Plos Med..

[32] Liberati A, Altman DG, Tetzlaff J, Mulrow C, Gøtzsche PC, Ioannidis JP (2009). The PRISMA statement for reporting systematic reviews and meta-analyses of studies that evaluate health care interventions: explanation and elaboration. J Clin Epidemiol..

[33] Cohen J (1960). A coefficient of agreement for nominal scales. Educ Psychol Meas..

[34] Moher D, Hopewell S, Schulz KF, Montori V, Gøtzsche PC, Devereaux PJ (2010). CONSORT 2010 Explanation and elaboration: updated guidelines for reporting parallel group randomised trials. BMJ.

[35] Von Elm E, Altman DG, Egger M, Pocock SJ, Gøtzsche PC (2007). The Strengthening the Reporting of Observational Studies in Epidemiology (STROBE) statement. Epidemiology.

[36] Liman TG, Zangemeister WH. Scanpath eye movements during visual mental imagery in a simulated hemianopia paradigm. J Eye Movement Res. 2012;5(1). 10.16910/jemr.5.1.2.

[37] Nowakowska A, Clarke ADF, Sahraie A, Hunt AR (2016). Inefficient search strategies in simulated hemianopia. J Exp Psychol.

[38] Nowakowska A, Clarke ADF, Sahraie A, Hunt AR (2019). Practice-related changes in eye movement strategy in healthy adults with simulated hemianopia. Neuropsychol..

[39] Schuett S, Kentridge RW, Zihl J, Heywood CA (2009). Are hemianopic reading and visual exploration impairments visually elicited? New insights from eye movements in simulated hemianopia. Neuropsychol..

[40] Schuett S, Kentridge RW, Zihl J, Heywood CA (2009). Adaptation of eye-movements to simulated hemianopia in reading and visual exploration: transfer or specificity?. Neuropsychol..

[41] Schuett S, Kentridge RW, Zihl J, Heywood CA (2009). Is the origin of the hemianopic line bisection error purely visual? Evidence from eye movements in simulated hemianopia. Vis Res..

[42] Zangemeister WH, Utz P (2002). An increase in a virtual hemianopic field defect enhances the efficiency of secondary adaptive gaze strategies. Cahiers Psychol Cog..

[43] Alberti CF, Goldstein RB, Peli E, Bowers AR (2017). Driving with Hemianopia v: do individuals with hemianopia spontaneously adapt their gaze scanning to differing hazard detection demands?. Trans Vis Sci Tech..

[44] Bahnemann M, Hamel J, De Beukelaer S, Ohl S, Kehrer S, Audebert H (2015). Compensatory eye and head movements of patients with homonymous hemianopia in the naturalistic setting of a driving simulation. J Neurol..

[45] Bowers AR, Ananyev E, Mandel AJ, Goldstein RB, Peli E (2014). Driving with hemianopia: IV. Head scanning and detection at intersections in a simulator. Invest Ophthalmol Vis Sci..

[46] Cazzoli D, Hopfner S, Preisig B, Zito G, Vanbellingen T, Jäger M, Nef T, Mosimann U, Bohlhalter S, Müri RM, Nyffeler T (2016). The influence of naturalistic, directionally non-specific motion on the spatial deployment of visual attention in right-hemispheric stroke. Neuropsychol..

[47] Fourtassi M, Rode G, Tilikete C, Pisella L (2016). Spontaneous ocular positioning during visual imagery in patients with hemianopia and/or hemineglect. Neuropsychol..

[48] Gbadamosi J, Zangemeister WH (2001). Visual imagery in hemianopic patients. J Cog Neurosci..

[49] Grasso PA, Làdavas E, Bertini C (2016). Compensatory recovery after multisensory stimulation in hemianopic patients: behavioral and neurophysiological components. Front Syst Neurosci..

[50] Hardiess G, Papageorgiou E, Schiefer U, Mallot HA (2010). Functional compensation of visual field deficits in hemianopic patients under the influence of different task demands. Vis Res..

[51] Kasneci E, Sippel K, Aehling K, Heister M, Rosenstiel W, Schiefer U, Papageorgiou E (2014). Driving with binocular visual field loss? A study on a supervised on-road parcours with simultaneous eye and head tracking. Plos One..

[52] Kasneci E, Sippel K, Heister M, Aehling K, Rosenstiel W, Schiefer U, Papageorgiou E (2014). Homonymous visual field loss and its impact on visual exploration: a supermarket study. Trans Vis Sci Tech..

[53] Keller I, Lefin-Rank G (2010). Improvement of visual search after audiovisual exploration training in hemianopic patients. Neurorehab Neural Rep..

[54] Levy-Bencheton D, Pelisson D, Prost M, Jacquin-Courtois S, Salemme R, Pisella L (2015). The effects of short-lasting anti-saccade training in homonymous hemianopia with and without saccadic adaptation. Front Behav Neurosci..

[55] Iorizzo DB, Riley ME, Hayhoe M, Huxlin KR (2011). Differential impact of partial cortical blindness on gaze strategies when sitting and walking - an immersive virtual reality study. Vis Res..

[56] Machner B, Sprenger A, Kömpf D, Sander T, Heide W, Kimmig H, Helmchen C (2009). Visual search disorders beyond pure sensory failure in patients with acute homonymous visual field defects. Neuropsychol..

[57] Machner B, Sprenger A, Sander T, Heide W, Kimmig H, Helmchen C, Kömpf D (2009). Visual search disorders in acute and chronic homonymous hemianopia: lesion effects and adaptive strategies. Ann NY Acad Sci..

[58] Mannan SK, Pambakian ALM, Kennard C (2010). Compensatory strategies following visual search training in patients with homonymous hemianopia: an eye movement study. J Neurol..

[59] Papageorgiou E, Hardiess G, Mallot HA, Schiefer U (2012). Gaze patterns predicting successful collision avoidance in patients with homonymous visual field defects. Vis Res..

[60] Passamonti C, Bertini C, Làdavas E (2009). Audio-visual stimulation improves oculomotor patterns in patients with hemianopia. Neuropsychol..

[61] Reinhard JI, Damm I, Ivanov IV, Trauzettel-Klosinski S (2014). Eye movements during saccadic and fixation tasks in patients with homonymous hemianopia. J Neuro-Ophthalmol..

[62] Roth T, Sokolov AN, Messias A, Roth P, Weller M, Trauzettel-Klosinski S (2009). Comparing explorative saccade and flicker training in hemianopia: a randomized controlled study. Neurol..

[63] Schoepf D, Zangemeister WH (1996). Target predictability influences the distribution of coordinated eye- head gaze saccades in patients with homonymous hemianopia. Neurol Res..

[64] Tant MLM, Cornelissen FW, Kooijman AC, Brouwer WH (2002). Hemianopic visual field defects elicit hemianopic scanning. Vis Res..

[65] Turton AJ, Angilley J, Longley V, Clatworthy P, Gilchrist ID (2018). Search training for people with visual field loss after stroke: a cohort study. Br J Occ Ther..

[66] Wood JM, McGwin G, Elgin J, Vaphiades MS, Braswell RA, DeCarlo DK (2011). Hemianopic and quadrantanopic field loss, eye and head movements, and driving. Invest Ophthalmol Vis Sci..

[67] Zangemeister WH, Meienberg O, Stark L, Hoyt WF (1982). Eye-head coordination in homonymous hemianopia. J Neurol..

[68] Zihl J (1999). Oculomotor scanning performance in subjects with homonymous visual field disorders. Vis Imp Res..

[69] Weiskrantz L. Blindsight: a case study and implications. Oxford: Oxford University Press; 1986.

