# Supplementary material for: Biomechanical adaptation to post-stroke visual field loss: a systematic review
Source: Syst Rev. 2021 Mar 27;10:84. doi: 10.1186/s13643-021-01634-4 (PMC8004433; doi:10.1186/s13643-021-01634-4)
Supplement: Supplementary file 2 — Additional file 2. [file 13643_2021_1634_MOESM2_ESM.docx]

supplementary table 1: Quality assessment of observational studies using the STROBE checklist

|  | Title  Abstract | Background | Objectives | Study  design | Setting | Participants | Variables | Measurement | Bias | Study  size | Quantitative  variables | Statistical  methods | Participants | Descriptive  data | Outcome  data | Main  results | Other analyses | Key  results | Limitations | Interpretation | Generalizability | Funding | Score |
| --- | --- | --- | --- | --- | --- | --- | --- | --- | --- | --- | --- | --- | --- | --- | --- | --- | --- | --- | --- | --- | --- | --- | --- |
| Alberti 2017 | + | + | + | + | + | + | + | + | - | - | + | + | - | + | + | + | n/a | + | - | + | + | + | 81 |
| Bahnemann 2015 | + | + | + | + | + | + | + | + | - | - | + | + | - | + | + | + | n/a | + | - | + | - | + | 76 |
| Bowers 2014 | + | + | + | + | + | + | + | + | - | - | + | + | + | + | + | + | n/a | + | + | + | - | + | 81 |
| Cazzoli 2016 | + | + | + | + | + | + | + | + | - | - | + | + | - | + | + | + | n/a | + | - | + | + | + | 81 |
| Fourtassi  2016 | + | + | + | + | + | + | + | + | - | - | + | + | - | + | + | + | n/a | + | - | + | - | - | 71 |
| Gbadamosi 2001 | + | + | + | + | + | + | + | + | - | - | + | + | - | + | + | + | n/a | + | - | + | - | - | 76 |
| Grasso et al 2016 | + | + | + | + | + | + | + | + | - | - | + | + | - | + | + | + | n/a | + | - | + | - | + | 76 |
| Hardiess 2010 | + | + | + | + | + | + | + | + | - | - | + | + | + | + | + | + | n/a | + | + | + | + | + | 90 |
| Iorizzo 2011 | + | + | + | + | + | + | + | + | - | - | + | + | + | + | + | + | n/a | + | - | + | - | + | 81 |
| Kasneci 2014a | + | + | + | + | + | + | + | + | - | - | + | + | - | + | + | + | n/a | + | + | + | - | + | 81 |
| Kasneci 2014b | + | + | + | + | + | + | + | + | - | - | + | + | - | + | + | + | n/a | + | + | + | - | + | 81 |
| Lévy-Bencheton et al 2015 | + | + | + | + | + | + | + | + | - | - | + | + | + | + | + | + | n/a | + | - | + | + | + | 85 |
| Liman 2012 | + | + | + | + | + | + | + | + | - | - | + | + | - | + | + | + | n/a | + | - | + | - | - | 71 |
| Machner  2009 | + | + | + | + | + | + | + | + | - | - | + | + | - | + | + | + | n/a | + | - | + | - | - | 71 |
| Machner 2009 | + | + | + | + | + | + | + | + | - | - | + | + | - | + | + | + | n/a | + | - | + | - | + | 76 |
| Mannan et al 2010 | + | + | + | + | + | + | + | + | - | - | + | + | + | + | + | + | n/a | + | - | + | + | + | 85 |
| Martin  2007 | + | + | + | + | + | + | + | + | - | - | + | + | + | + | + | + | n/a | + | - | + | - | + | 81 |
| Nowakowsk2016 | + | + | + | + | + | + | + | + | - | - | + | + | + | + | + | + | n/a | + | - | + | - | + | 81 |
| Nowakowsk2019 | + | + | + | + | + | + | + | + | - | - | + | + | - | + | + | + | n/a | + | + | + | + | - | 81 |
| Pambakian  2000 | + | + | + | + | + | + | + | + | - | - | + | + | - | + | + | + | n/a | + | + | + | - | + | 81 |
| Papageorgiou 2012 | + | + | + | + | + | + | + | + | - | - | + | + | + | + | + | + | n/a | + | - | + | + | + | 85 |
| Passamonti et al 2009 | + | + | + | + | + | + | + | + | - | - | + | + | + | + | + | + | n/a | + | - | + | - | + | 81 |
| Reinhard 2014 | + | + | + | + | + | + | + | + | - | - | + | + | - | + | + | + | n/a | + | - | + | - | + | 76 |
| Schoepf 1996 | + | + | + | + | + | + | + | + | - | - | + | + | - | + | + | + | n/a | + | - | + | - | - | 71 |
| Schuett 2009a | + | + | + | + | + | + | + | + | - | - | + | + | - | + | + | + | + | + | - | + | - | + | 76 |
| Schuett 2009b | + | + | + | + | + | + | + | + | - | - | + | + | - | + | + | + | + | + | - | + | - | + | 76 |
| Schuett 2009c | + | + | + | + | + | + | + | + | - | - | + | + | - | + | + | + | + | + | - | + | - | + | 76 |
| Tant 2002 | + | + | + | + | + | + | + | + | - | - | + | + | - | + | + | + | n/a | + | - | + | + | - | 76 |
| Turton et al 2008 | + | + | + | + | + | + | + | + | - | - | + | - | + | + | + | + | n/a | + | + | + | - | + | 81 |
| Wood 2011 | + | + | + | + | + | + | + | + | - | - | + | + | + | + | + | + | n/a | + | + | + | + | + | 90 |
| Zangemeister 2002 | + | + | + | + | + | + | + | + | - | - | + | + | - | + | + | + | n/a | + | - | + | - | - | 71 |
| Zihl 1995 | + | + | + | + | + | + | + | + | - | - | + | + | - | + | + | + | n/a | + | - | + | + | - | 76 |
| Zihl 1999 | + | + | + | + | + | + | + | + | - | - | + | + | - | + | + | + | n/a | + | - | + | - | + | 76 |
