# Supplementary material for: Biomechanical adaptation to post-stroke visual field loss: a systematic review
Source: Syst Rev. 2021 Mar 27;10:84. doi: 10.1186/s13643-021-01634-4 (PMC8004433; doi:10.1186/s13643-021-01634-4)
Supplement: Supplementary file 3 — Additional file 3. [file 13643_2021_1634_MOESM3_ESM.docx]

Supplementary table 2: Quality assessment of randomised trials using the CONSORT checklist

| **Section/Topic** | **Checklist item** | **Item No** | **Keller 2010** | **Roth 2009** | **Schuett 2009** |
| --- | --- | --- | --- | --- | --- |
| Title and abstract | Identification as a randomised trial in the title | 1a | - | + | - |
|  | Structured summary of trial design, methods, results, and conclusions | 1b | + | + | - |
| Introduction | Objectives | 2b | + | + | + |
| Methods | Trial design | 3a | + | + | + |
|  | Changes to methods | 3b | - | - | - |
|  | Eligibility | 4a | + | + | + |
|  | Interventions for each group | 5 | + | + | + |
|  | Outcome measures | 6a | + | + | + |
|  | Changes to trial outcomes | 6b | - | - | - |
|  | Sample size | 7a | - | - | - |
|  | Interim analysis | 7b | N/A | N/A | N/A |
|  | Method of random allocation sequence | 8a | + | - | + |
|  | Randomisation | 8b | + | - | + |
|  | Implementation of random allocation | 9 | + | - | + |
|  | Generation of random allocation | 10 | - | - | - |
|  | Blinding | 11a | + | - | - |
|  | Similarity of interventions | 11b | + | + | + |
|  | Statistical methods | 12a | + | + | + |
|  | Additional analyses | 12b | + | + | + |
| Results | No. of participants | 13a | + | + | + |
|  | Losses and exclusions | 13b | + | + | - |
|  | Dates of recruitment - follow up | 14a | + | + | + |
|  | Reason trial ended | 14b | N/A | N/A | N/A |
|  | Baseline demographic | 15 | + | + | + |
|  | Analysis of original assigned groups | 16 | + | + | + |
|  | Results with precision | 17a | + | + | + |
|  | Additional analysis | 18 | + | + | + |
|  | Harms | 19 | - | - | - |
| Discussion | Limitations | 20 | + | - | + |
|  | Generalisability | 21 | + | + | + |
|  | Consistent interpretation | 22 | + | + | + |
| Other Info | Registration | 23 | - | - | - |
|  | Access to full protocol | 24 | - | - | - |
|  | Funding | 25 | + | - | - |
| Overall % |  |  | 81 | 59 | 62 |
